# Multi-Swarm Algorithm for Extreme Learning Machine Optimization

**DOI:** 10.3390/s22114204

**Published:** 2022-05-31

**Authors:** Nebojsa Bacanin, Catalin Stoean, Miodrag Zivkovic, Dijana Jovanovic, Milos Antonijevic, Djordje Mladenovic

**Affiliations:** 1 Faculty of Informatics and Computing, Singidunum University, Danijelova 32, 11010 Belgrade, Serbia; mzivkovic@singidunum.ac.rs (M.Z.); mantonijevic@singidunum.ac.rs (M.A.); 2 Romanian Institute of Science and Technology, 400022 Cluj-Napoca, Romania; catalin.stoean@rist.ro; 3 College of Academic Studies “Dositej”, Bulevar Vojvode Putnika 7, 11000 Belgrade, Serbia; dijana.jovanovic@akademijadositej.edu.rs (D.J.); djordjemladenovic@akademijadositej.edu.rs (D.M.)

**Keywords:** machine learning, extreme learning machine, meta-heuristic algorithms, swarm intelligence, multi-swarm algorithm, hybridization

## Abstract

There are many machine learning approaches available and commonly used today, however, the extreme learning machine is appraised as one of the fastest and, additionally, relatively efficient models. Its main benefit is that it is very fast, which makes it suitable for integration within products that require models taking rapid decisions. Nevertheless, despite their large potential, they have not yet been exploited enough, according to the recent literature. Extreme learning machines still face several challenges that need to be addressed. The most significant downside is that the performance of the model heavily depends on the allocated weights and biases within the hidden layer. Finding its appropriate values for practical tasks represents an NP-hard continuous optimization challenge. Research proposed in this study focuses on determining optimal or near optimal weights and biases in the hidden layer for specific tasks. To address this task, a multi-swarm hybrid optimization approach has been proposed, based on three swarm intelligence meta-heuristics, namely the artificial bee colony, the firefly algorithm and the sine–cosine algorithm. The proposed method has been thoroughly validated on seven well-known classification benchmark datasets, and obtained results are compared to other already existing similar cutting-edge approaches from the recent literature. The simulation results point out that the suggested multi-swarm technique is capable to obtain better generalization performance than the rest of the approaches included in the comparative analysis in terms of accuracy, precision, recall, and f1-score indicators. Moreover, to prove that combining two algorithms is not as effective as joining three approaches, additional hybrids generated by pairing, each, two methods employed in the proposed multi-swarm approach, were also implemented and validated against four challenging datasets. The findings from these experiments also prove superior performance of the proposed multi-swarm algorithm. Sample code from devised ELM tuning framework is available on the GitHub.

## 1. Introduction

Extreme machine learning (ELM) represents one of the recent and promising approaches that can be applied to the single hidden layer feed-forward artificial neural networks (SLFN). This approach was initially proposed in [1], and it introduced the concept that the input weight and bias values in the hidden layer are allocated in a random fashion, while the output weight values are computed by utilizing the Moore–Penrose (MP) pseudo inverse [2]. ELMs have shown excellent generalization capabilities [3], and they are known to be very fast and efficient due to the fact that they do not require traditional training, which is one of the most time-consuming tasks when dealing with other types of neural networks. By different training, we mean that ELM models learn without tuning hidden parameters in several iterations, and the only parameter that needs to be determined is the weight between the hidden layer and the output layer, using MP, as mentioned above.

ELMs require an adequate number of neurons in the hidden layer in order to obtain good performance and fast convergence. The difference between ELMs and other traditional machine learning (ML) models that typically utilize the gradient-descent-based algorithms is that ELMs use randomly allocated input weight and bias values that do not alter during the learning process. This approach prevents some of the issues that commonly accompany the gradient-descent-methods, such as iterative tuning of the weight and bias values, lingering in the local minimums, and slowing converging speed. Nevertheless, the appropriate number of neurons that make the hidden layer still remains one of the open questions that ELMs face.

Several enhanced ELM variants were proposed subsequently, most of which deal with the appropriate number of hidden neurons. The authors in [4] have introduced the pruned extreme learning machine (P-ELM) and used it for classifying patterns. P-ELM starts with a large number of neurons in a hidden layer, and utilizes statistical methods for determining the relevance of the neurons based on the class labels. The neurons that have been determined to be irrelevant are removed from the network, thus narrowing down the total number of neurons. Evolutionary ELM (E-ELM) that was proposed by [5] optimizes the input weight and bias values by applying the differential evolution method, and calculates the outputs with MP general inverse. The enhanced variant of the E-ELM suggested by [6], i.e., the self-adaptive evolutionary ELM (SaE-ELM), uses a self-adaptive differential evolution algorithm for hidden parameters’ optimization, and determines the output weight values analytically. The optimally-pruned ELM approach (OP-ELM), developed by [7], tackles the problem of a large number of hidden neurons by introducing neuron ranking. OP-ELM also begins with a large number of neurons as standard ELM approach, and narrows it down by utilizing the multi-response sparse regression algorithm (MRSR) for calculating ranks of neurons, and leave-one-out (LOO) validating technique for determining the optimal number of neural cells.

Another approach named incremental ELM (I-ELM) was suggested by [8] and proposed adding neurons one at the time to the hidden layer and calculating the training rate after every single added cell. The process halts either when the maximal amount of neurons is met, or when the training rate starts decreasing. More recent research published by [2] proposed two swarm intelligence meta-heuristics to optimize the ELM, namely ELM-ABC (using the artificial bee colony meta-heuristics) and ELM-IWO (based on the invasive weed optimization method). Swarm meta-heuristics are used for tuning the input weight and bias parameters, while the ELM calculates the output weight values in the standard, analytical way.

As previously stated, the ELM performance mostly depends on the number of neurons in the hidden layer and the initialized weights between input features and each neuron in the hidden layer. The extensive literature survey showed that meta-heuristics-based approaches to ELM optimization are scarce and insufficiently exploited in this domain, despite the very promising results obtained in other ML domains. This paper proposes weights and biases optimization in the ELM model by a multi-swarm algorithm. A straightforward heuristic is used to determine the promising number of neurons in the hidden layer.

The proposed approach utilizes a novel multi-swarm hybrid algorithm that combines three well-known swarm intelligence meta-heuristics, namely the artificial bee colony algorithm (ABC), the firefly algorithm (FA), and the sine–cosine algorithm (SCA). The algorithm is a high- and low-level combination of hybridized algorithms that exploits their strengths and overcomes deficiencies of each individual one. The main motivation behind this research lies in the fact that ELMs are efficient, fast, they do not require training, and, on the other hand, they have not been exploited sufficiently, especially in combination with meta-heuristics. The obtained results are comparable with the results of other ML methods that require training and significantly more time for execution. Inspired by the experiments given in [2], the proposed method has been tested on seven benchmark datasets in order to provide a fair comparison of the results.

Moreover, to prove that combining two algorithms is not as effective as joining three approaches, in an additional set of experiments, hybrids generated by pairing each two methods employed in the proposed multi-swarm approach, were also implemented and validated against four imbalanced challenging datasets.

The rest of the manuscript is structured in the following way. Section 2 provides a literature survey on ELM and swarm intelligence meta-heuristics. The description of the proposed multi-swarm approach is provided in Section 3. Section 4 describes the conducted experiments and exhibits the simulation findings on seven datasets together with the comparative analysis with similar approaches. Lastly, Section 5 delivers final observations, proposes future directions in this area, and concludes the paper.

## 2. Background

This section first introduces the ELM as one of the ML models. After that, a brief survey of swarm intelligence meta-heuristics is provided, together with the most common applications. Finally, an overview of the ELM models optimized with swarm intelligence meta-heuristic algorithms is given.

### 2.1. Extreme Learning Machine

Extreme learning machine (ELM) was proposed by Huang et al. [1] for single-hidden layer feed-forward neural networks (SLFNs). The algorithm randomly chooses the input weights and analytically determines the output weights of SLFNs. After the input weights and the hidden layer biases are chosen arbitrarily, SLFNs can be simply considered as a linear system and the output weights of SLFNs can he analytically determined through a simple generalized inverse operation of the hidden layer output matrices. This algorithm provides a faster learning speed than traditional feed-forward network learning algorithms, while obtaining better generalization performance. Additionally, ELM tends to reach the smallest training error and the smallest norm of weights. The output weights are computed using Moore–Penrose (MP) generalized inverse [9]. As shown in [10], the learning speed of ELM can be thousands of times faster than conventional learning algorithms with better generalization performance than the gradient-based learning models. Unlike the traditional classic gradient-based learning algorithms that only work for differentiable activation functions, the ELM learning algorithm could be used to train SLFNs with many non-differentiable activation functions.

For a set of training samples ${\left\{\left(x_{j},t_{j}\right)\right\}}_{j=1}^{N}$ with *N* samples and m classes, the SLFN with L hidden nodes and activation function g(x) is expressed as in (1) [1], where $w_{i}={\left[w_{i1},\ldots ,w_{in}\right]}^{T}$ is the input weight, $b_{i}$ is the bias of the *i*th hidden node, $\beta_{i}={\left[\beta_{i1},\ldots ,\beta_{im}\right]}^{T}$ is the weight vector connecting the *i*th hidden node and the output nodes, $w_{i}\cdot x_{j}$ is inner product of $w_{i}$ and $x_{j}$ and $t_{j}$ is network output with respect to input $x_{j}$.

(1) $$\sum_{i=1}^{L}\beta_{i}g\left(w_{j}\cdot x_{j}+b_{i}\right)=t_{j},j=1,2,\ldots ,N$$

The Equation (1) can be written as:

(2) Hβ=T

where

(3) $$H={\left[\begin{array}{ccc} g(w_{1}\cdot x_{1}+b_{1}) & \ldots & g(w_{L}\cdot x_{1}+b_{L})\\ \vdots & \ldots & \vdots \\ g(w_{1}\cdot x_{N}+b_{1}) & \ldots & g(w_{L}\cdot x_{N}+b_{L}) \end{array}\right]}_{NxL},\beta ={\left[\begin{array}{c} \beta_{1}^{T}\\ \vdots \\ \beta_{L}^{T} \end{array}\right]}_{Lxm},T={\left[\begin{array}{c} t1^{T}\\ \vdots \\ tN^{T} \end{array}\right]}_{Nxm}$$

In this equation, *H* is the hidden layer output matrix of the neural network as explained in [11], while β is the output weight matrix.

The ELM is successfully used in solving many practical problems, such as text categorization [12], face recognition [13], image classification [14], different medical diagnostics [15,16], and so on. Over time, researchers have presented various improvements for the original ELM. In [4], authors propose a pruned ELM algorithm as a systematic and automated approach for designing an ELM classifier network. By considering the relevance of the hidden nodes to the class labels, the algorithm removes the irrelevant nodes from the initial large set of hidden nodes. Zhu et al. in their paper [5], presented an improved ELM, which uses the differential evolutionary algorithm to tune the input weights and MP generalized inverse. Experimental results show that this approach provides good generalization performance with more compact networks. Adopting this idea, in [17], the authors introduced a new kind of evolutionary algorithm based on PSO which, using the concepts of ELM, can train the network more suitably for some prediction problems. In order to deal with data with imbalanced class distribution, in [18], a weighted ELM is proposed which is able to generalize to balanced data. Recently, Alshamiri et al. presented in [2] a model for tuning ELM by using two SI based techniques—ABC and Invasive Weed Optimization (IWO) [19]. In this approach, the input weights and hidden biases are selected using ABC and IWO and the output weights are computed using the MP generalized inverse.

### 2.2. Swarm Intelligence

Swarm intelligence (SI) is an artificial intelligence approach which is inspired by the natural behavior to solve optimization problems [20]. Over time, many different SI algorithms were developed, including ant colony optimization (ACO) [21], particle swarm optimization (PSO) [22], artificial bee colony (ABC) [23], the firefly algorithm (FA) [24], cuckoo search (CS) [25], the bat algorithm (BA) [26], the whale optimization algorithm (WOA) [27], elephant herding optimization (EHO) [28], and many others [29,30,31]. More recent, but successful, approaches include monarch butterfly optimization (MBO) [32], slime mould algorithm (SMA) [33], moth search algorithm (MSA) [34], hunger games search (HGS) [35], colony predation algorithm (CPA) [36]. Still, we do not claim the list above is exhaustive.

The algorithms from this group have been used in a wide spectrum of different challenges with NP-hardness from the computer science field. These applications include the problem of global numerical optimization [37], scheduling of tasks in the cloud-edge environments [38,39,40], health care systems and pollution prediction [41], the problems of wireless sensors networks including localization and lifetime maximization [42,43,44], artificial neural networks optimization [45,46,47,48,49,50,51,52,53,54,55,56,57], feature selection in general [58,59], text document clustering [48], cryptocurrency values prediction [60], computer-aided medical diagnostics [61,62,63,64], and, finally, the ongoing COVID-19 pandemic related applications [65,66,67].

### 2.3. ELM Tuning by Swarm Intelligence Meta-Heuristics

An extensive literature survey indicates that swarm intelligence meta-heuristics have not been sufficiently exploited for the optimization of the ELM. In addition to the already mentioned paper [2] that inspired this research, just a few approaches that combine ELM and meta-heuristics were published in the past several years. Research published in [68] proposed a hybrid PSO-ELM model, and used it for flash flood prediction. The algorithm was tested on the geospatial database of a typhoon area, and compared to traditional ML models. The obtained results have shown that the PSO-ELM model was superior to other ML models. ELM optimized by PSO was also used in [69], where the authors used ELM to derive hydropower reservoir operation rules. The proposed method was named class-based evolutionary extreme learning machine (CEELM), and it combined k-means clustering that was used to separate the influential factors into clusters with more simple pattern, followed by the application of the ELM optimized by PSO for identifying the complex input–output relationships for every cluster. According to the authors, CEELM showed excellent generalization capabilities.

Faris et al. [70] discussed the application of the salp swarm algorithm (SSA) for optimizing ELM and improving the accuracy. The proposed approach was tested against ten benchmark datasets and compared to other popular training techniques. They concluded that ELM hybridized with SSA outperforms other approaches in achieved accuracy, and obtained satisfactory prediction stability. Finally, improved bacterial foraging optimization algorithm (BFO) has been proposed in [71] and applied for the ELM optimizing task. The obtained results once again indicated that it is possible to achieve similar or even better performances than other ML methods, in a reduced amount of time.

## 3. Proposed Hybrid Meta-Heuristics

This section first introduces the basic implementations of the three algorithms used for the proposed research, namely ABC, FA, and SCA. Since each algorithm has specific deficiencies, a novel multi-swarm algorithm has been proposed, that combines the strengths of the individual algorithms and overcomes their individual flaws, by creating synergy and achieving a complementary effect.

### 3.1. Original Algorithms

#### 3.1.1. The Original ABC Algorithm

The artificial bee colony (ABC) algorithm was designed for continuous optimization problems and it was inspired by the foraging behavior of honey bees [23,72]. ABC uses three control parameters and utilizes three classes of artificial bees: employed bees, onlookers, and scouts. Employed bees make half of a colony. In this model, food source represents the possible problem solution. There is only one employed bee per each food source. The employed bee performs the search process by examining the solution’s neighborhood. The onlooker chooses a food source for exploitation based on the information which they gain from employed bees. If a food source does not improve for a predetermined number of cycles, the scouts replace that food source with a new one which is chosen randomly. The limit parameter controls this process [73].

The ABC algorithm, as an iterative algorithm, starts by associating each employed bee with a randomly generated food source. Each bee $x_{i}$(i=1,2,…,N) is a *D*-dimensional vector, where *N* denotes the size of the population. The initial population of candidate solutions is created using the following expression (4), where $x_{i,j}$ is the *j*-the parameter of th $i^{th}$ bee in the population, rand(0,1) is a random real number between 0 and 1, and $ub_{j}$ and $lb_{j}$ are upper and lower bounds of the $j^{th}$ parameter, respectively. Naturally, *x* represents a different element than the training samples from Equation (1).

(4) $$x_{i,j}=lb_{j}+rand\left(0,1\right)*\left(ub_{j}-lb_{j}\right),$$

There are many formulations of the fitness function, but in most implementations, for maximization problems, fitness is simply proportional to the value of objective function. In case the problem to be solved targets the minimization of a function denoted here by objFun, the task is converted for maximization using a modification, such as in (5).

(5) $$fitness_{i}=\left\{\begin{array}{c} \frac{1}{objFun_{i}},\;\;if\;objFun_{i}>0\\ 1+|objFun_{i}|,\;\;\mathrm{otherwise} \end{array}\right.$$

Each employed bee discovers a food source in its neighborhood and evaluates its fitness. The discovery of a new neighborhood solution is simulated with the expression (6), where $x_{i,j}$ is $j^{th}$ parameter of the old solution *i*, $x_{k,j}$ is $j^{th}$ parameter of a neighbor solution *k*, ϕ is a random number between 0 and 1, and MR is modification rate. MR is a control parameter of ABC algorithm.

(6) $$v_{i,j}=\left\{\begin{array}{c} x_{i,j}+\phi *\left(x_{i,j}-x_{k,j}\right),R_{j}<MR\\ x_{i,j},\;\;\mathrm{otherwise} \end{array}\right.$$

If the fitness of the new solution is higher than the fitness of the old one, the employed bee continues the exploitation process with the new food source, otherwise it retains the old one. Employed bees share information about the fitness of a food source with onlookers, and onlookers select a food source *i* with a probability that is proportional to the solution’s fitness:

(7) $$p_{i}=\frac{fitness_{i}}{\sum_{i=1}^{N}fitness_{i}}$$

#### 3.1.2. The Original Firefly Algorithm

The Firefly algorithm was introduced by Yang [24]. The proposed model uses brightness and attractiveness of fireflies. Brightness is determined by the objective function value, while attractiveness depend on the brightness. This is expressed with Equation (8) [24], where I(x) represents attractiveness and f(x) denotes the value of objective function at location *x*. Again, it is noted that the *x* in the current subsection should not be mistaken for the representations in the previous subsections.

(8) $$I\left(x\right)=\left\{\begin{array}{cc} \frac{1}{f(x)} & ,\;\mathrm{if}\;f(x)>0\\ 1+|f(x)| & ,\;\mathrm{otherwise}\; \end{array}\right.$$

The attractiveness of the firefly decreases, as the distance from the light source increases [24]:

(9) $$I\left(r\right)=\frac{I_{0}}{1+\gamma r^{2}}$$

where I(r) represents light intensity at distance *r*, and $I_{0}$ stands for the light intensity at the source. In order to model a real nature system, where the light is partially absorbed by its surroundings, the FA uses the γ parameter, which represents the light absorption coefficient. The combined effect of the inverse square law for distance and the γ coefficient is approximated with the following Gaussian form [24]:

(10) $$I\left(r\right)=I_{0}\cdot e^{-\gamma r^{2}}$$

Moreover, each firefly individual utilizes attractiveness β, which is directly proportional to the light intensity of a given firefly and also depends on the distance, as shown in Equation (11).

(11) $$\beta \left(r\right)=\beta_{0}\cdot e^{-\gamma r^{2}}$$

where parameter $\beta_{0}$ designates attractiveness at distance r=0. It should be noted that, in practice, Equation (11) is often replaced by Equation (12) [24]:

(12) $$\beta \left(r\right)=\frac{\beta_{0}}{1+\gamma r^{2}}$$

Based on the above, the basic FA search equation for a random individual *i*, which moves in iteration t+1 to a new location $x_{i}$ towards individual *j* with greater fitness, is given as [24]:

(13) $$x_{i}^{t+1}=x_{i}^{t}+\beta_{0}\cdot e^{-\gamma r_{i,j}^{2}}\left(x_{j}^{t}-x_{i}^{t}\right)+\alpha^{t}\left(\kappa -0.5\right)$$

where α stands for the randomization parameter, the random number drawn from Gaussian or a uniform distribution is denoted as κ, and $r_{i,j}$ represents the distance between two observed fireflies *i* and *j*. Typical values that establish satisfying results for most problems for $\beta_{0}$ and α are 1 and [0,1], respectively.

The $r_{i,j}$ is the Cartesian distance, which is calculated by using Equation (14).

(14) $$r_{i,j}=\left|\right|x_{i}-x_{j}\left|\right|=\sqrt{\sum_{k=1}^{D}{\left(x_{i,k}-x_{j,k}\right)}^{2}}$$

where *D* marks the number specific problem parameters.

#### 3.1.3. The Original SCA Method

The sine–cosine algorithm (SCA) proposed in Mirjalili [74] is based on mathematical model of the sine and cosine trigonometric functions. The solutions’ positions in the population are updated based on the sine and cosine functions outputs which makes them oscillate around the best solution. The return values of these functions are between −1 and +1, which is the mechanism that keeps the solutions fluctuating. An algorithm starts with generating a set of random candidate solutions within the boundaries of the search space in the initialization phase. Exploration and exploitation are controlled differently throughout the execution by random adaptive variables.

The solutions’ position update process is performed in each iteration by using Equations (15) and (16), where $X_{i}^{t}$ and $X_{i}^{t+1}$ is the current solution’s position in the *i*-th dimension at *t*-th and i+1-th iteration, respectively, $r_{1-3}$ are pseudo-randomly generated numbers, the $P_{i}^{*}$ denotes the destination point’s position (current best approximation of an optimum) in the *i*-th dimension, while symbol || represents the absolute value. The same notations as in the original manuscript where the method was initially proposed [74] are used in this manuscript.

(15) $$X_{i}^{t+1}=X_{i}^{t}+r_{1}\cdot sin\left(r_{2}\right)\cdot \left|r_{3}\cdot P_{i}^{*t}-X_{i}^{t}\right|$$

(16) $$X_{i}^{t+1}=X_{i}^{t}+r_{1}\cdot cos\left(r_{2}\right)\cdot \left|r_{3}\cdot P_{i}^{*t}-X_{i}^{t}\right|$$

These two equations are used in combination by using control parameter $r_{4}$:

(17) $$X_{i}^{t+1}=\left\{\begin{array}{c} X_{i}^{t+1}=X_{i}^{t}+r_{1}\cdot sin\left(r_{2}\right)\cdot \left|r_{3}\cdot P_{i}^{*t}-X_{i}^{t}\right|,\;r_{4}<0.5\\ X_{i}^{t+1}=X_{i}^{t}+r_{1}\cdot cos\left(r_{2}\right)\cdot \left|r_{3}\cdot P_{i}^{*t}-X_{i}^{t}\right|,\;r_{4}\geq 0.5, \end{array}\right.$$

where $r_{4}$ represents a randomly generated number between 0 and 1.

It is noted that, for every component of each solution in the population, new values for pseudo-random parameters $r_{1-4}$ are generated.

The algorithm’s search process is controled by four random parameters and they influence the current and the best solution’s positions. In order to converge towards the global optima, the balance between solutions is required. This is achieved by changing the range of the based functions in an ad-hoc manner. Exploitation is guaranteed by the fact that sine and cosine functions exhibit cyclic patterns which allow for reposition around the solution. Changes in ranges of sine and cosine functions allow the algorithm to search outside of their corresponding destinations. Furthermore, the solution requires its position not to overlap with the areas of other solutions.

For better quality of randomness, the values for parameter $r_{2}$ are generated within the range [0,2Π] and that guarantees exploration. The controls of the balance between diversification and exploitation are shown with Equation (18).

(18) $$r_{1}=a-t\frac{a}{T},$$

where *t* is the current iteration, *T* represents the maximum number of iterations in a run, while *a* is a constant.

### 3.2. Proposed Multi-Swarm Meta-Heuristics Algorithm

#### 3.2.1. Motivation and Preliminaries

The effectiveness of meta-heuristics in optimization process largely depends on efficiency and balance between exploitation and exploration, that direct the search towards optimum (sub-optimum) solutions. Additionally, according to the no free lunch theorem (NFL), optimizer without flaws does not exist, nor there is one which can render satisfying solutions for all kinds of NP-hard challenges. Therefore, every meta-heuristics suffers from some deficiencies and also each one has distinctive advantages over others.

One of promising techniques that can be used to combine different meta-heuristics is hybridization [75,76]. If the right meta-heuristics are chosen as components of hybrid method, strengths of one approach compensates weaknesses of the other, and vice-versa. Hybrid meta-heuristics are proven as efficient optimizers and they were validated against different problems [56,59,77,78,79].

In the modern literature, many taxonomies of hybrid meta-heuristics can be found, however on of the most widely adopted is the one provided by Talbi [76]. According to [76], by using the notion of hierarchical classification, hybrid algorithms can be differentiated between low-level (LLH) and high-level hybrids (HLH). In the case of LLH, search function of one method is replaced with one that belongs to other optimization method. Conversely, HHL approaches are self-contained [76].

Further, both LLH and HLH can be executed in relay or teamwork mode [76]. The first mode executes in a pipeline manner, where the output of first meta-heuristics is used as the input for the second, while in the case of teamwork mode, meta-heuristics evolve in parallel, cooperatively exploring and exploiting search space.

Approach which is developed for the purpose of this research represents combination of LLH and HLH and encompasses well-known ABC, FA, and SCA meta-heuristics. These three meta-heuristics are chosen due to its complementary weaknesses and strengths that makes them a promising candidates for hybridization.

Based on the previous findings, the ABC algorithm has efficient exploration mechanism which discards individuals that can not be improved in the predefined number of iterations, however it suffers from poor exploitation [73]. Conversely, both FA and SCA meta-heuristics exhibit above average intensification abilities, but they do not employ explicit exploration mechanism which leads to lower diversification capabilities [67,80]. Dynamic FA implementation controls exploitation–exploration balance by shrinking parameter α throughout iterations, while the SCA also uses dynamic parameter $r_{1}$. However, if the initially generated population is far away from optimum, dynamic parameters would only perform exploration around current solutions (novel solutions from other regions of the search space will not be generated), and when termination condition is reached, in most cases local optimum solutions will be rendered. Additionally, regardless of good intensification of FA and SCA, the search can be further boosted by combination of its search expressions. This stems from the fact that FA and SCA employ different search equations—the FA uses the notion of distance between solution, while the SCA employs trigonometric functions.

Motivated by the facts provided above, proposed hybrid meta-heuristics first combines FA and SCA algorithms in a form of LLH with teamwork mode and afterwards such approach is hybridized with the ABC meta-heuristics, forming a HLH teamwork mode optimizer. Method which is proposed for the purpose of this research is therefore named multi-swarm-ABC-FA-SCA (MS-AFS).

#### 3.2.2. Overview of MS-AFS

In addition to combining ABC, FA, and SCA meta-heuristics, proposed MS-AFS also employs the following mechanisms:

- Chaotic and quasi-reflection-based learning (QRL) population initialization in order to establish boosting of the search by redirecting solutions towards more favorable parts of the domain;
- Efficient learning mechanism between swarms with the goal of combining weakness and strengths of different approaches more efficiently.

The concept of employing chaotic maps in meta-heuristics methods was first proposed by Caponetto et al. in [81]. The stochastic essence of the majority of meta-heuristics methods relates on random number generators. Nevertheless, several recent studies suggest that the search procedure could be improved if it were grounded in chaotic sequences [82,83].

Numerous chaotic maps exist, including circle, Chebyshev, logistic, sine, sinusoidal, tent, and many others. Extensive simulations conducted for the purpose of current, as well as previous research [63] with all the above-mentioned maps yielded the conclusion that the best results can be obtained by applying the logistic map, that was selected for implementation.

To establish chaotic-based population initialization, pseudo-random number $\theta_{0}$ is generated, as the seed for chaotic sequence θ created by the logistic mapping:

(19) $$\theta_{i+1}=\mu \theta_{i}\times \left(1-\theta_{i}\right),i=1,2,\ldots ,N-1,$$

where *N* denotes the population size, *i* is the sequence number, while μ is chaotic sequence control parameter. The μ was set to 4, as suggested in [84], while $0<\theta_{0}<1$ and $\theta_{0}\neq 0.25,0.5,0.75,1$.

Every parameter *j* of each solution *i* is mapped to rendered chaotic sequences by the following equation:

(20) $$X_{i}^{c}=\theta_{i}X_{i},$$

where $X_{i}^{c}$ is new position of individual *i* after chaotic perturbations.

The QRL procedure was initially proposed in [85]. This approach implies the generation of the quasi-reflexive-opposite solutions following the logic that if the original individual is positioned at a large distance from the optimum, a decent chance exists that the opposite solution could be located much nearer to the optimum.

When utilizing the QRL procedure described above, the quasi-reflexive-opposite individual $X^{qr}$ of the solution *X* will be created by applying the following expression for every component *j* of solution *X*:

(21) $$X^{qr}=\mathrm{rnd}\left(\frac{LB+UB}{2},X\right),$$

where $\mathrm{rnd}\left(\frac{LB+UB}{2},X\right)$ is used to generate an arbitrary number from the uniform distribution within $\left[\frac{LB+UB}{2},X\right]$, and LB and UB are lower and upper search boundaries, respectively. This strategy will be executed for each parameter of observed solution *X* in *D* dimensions.

Taking all into account, population initialization of proposed MS-AFS is summarized in Algorithm 1.

As it can be observed from Algorithm 1, the size of starting population $P_{start}$ is N/2 individuals. In this way, the fitness function evaluations FFEs in the initialization phase are executed only *N* times and additional load, in terms of computational requirements, on the MS-AFS complexity is not imposed.

After initialization of population *P* by Algorithm 1, N/2 worse solutions are chosen as the initial population ($P_{1}$) for first swarm ($s_{1}$), while remaining individuals ($P_{2}$) are delegated to the second swarm ($s_{2}$). The $s_{2}$ is created by establishing LLH with teamwork mode between FA and SCA algorithms, while the $s_{1}$ is executed only by the ABC meta-heuristics. Due to the fact that the ABC exhibits better exploration abilities and that it has more chance to hit the favorable regions of the search domain, worse N/2 individuals are chosen as initial population for the $s_{1}$.
**Algorithm 1** Pseudo-code for chaotic and QRL population initializationStep 1: Generate starting population $P_{start}$ of N/2 solutions with standard initialization expression: $X_{i}=LB+\left(UB-LB\right)\cdot rand\left(0,1\right),\;\;i=1,\ldots ,N/2$, where rand(0,1) represents pseudo-random number drawn from the range [0,1].Step 2: Randomly select 2 subsets of N/4 from $P_{start}$ for chaotic and QRL initialization, denoted as $P^{c}$ and $P^{qrl}$, respectively.Step 3: Extend $P^{c}$ by applying chaotic sequences to each individual in $P^{c}$ using expressions (19) and (20). The size of $P^{c}$ after extension is N/2.Step 4: Extend $P^{qrl}$ by applying QRL mechanism to each individual in $P^{qrl}$ using expression (21). The size of $P^{qrl}$ after extension is N/2.Step 5: Calculate fitness of all individuals from $P^{c}$ and $P^{qrl}$.Step 6: Sort all solutions from $P^{c}\cup P^{qrl}$ according to fitness.Step 7: Select *N* best solutions as the initial population *P*.

The $s_{2}$ simply combines search expressions of FA and SCA algorithms, Equations (13) and (17), respectively, and in each iteration every individual is evolved either by performing FA or SCA search. Finally, the $s_{1}$ and $s_{2}$ execute independently, where each swarm evolves its own population of candidate solutions.

The $s_{1}$ and $s_{2}$ search processes are shown in Algorithms 2 and 3, respectively.
**Algorithm 2** Search process of *s*_1_—ABC algorithm**for** each solution $X_{i}$ **do**   perform employed bee phase according to Equation (6)   perform onlooker bee phase according to expressions (6) and (7)**end for**perform scout bee phase (explicit exploration) according to expression (4)

**Algorithm 3** Search process of *s*_2_—LLH between FA and SCA
**for** each solution $X_{i}$ **do**   **if** rand(0,1)>0.5 **then**     Evolve $X_{i}$ by FA search—expression (13)   **else**     Evolve $X_{i}$ by SCA search—expression (17)   **end if**
**end for**



However, as noted above, in order to facilitate the search, after ψ iterations, the mechanism of exchanging knowledge (knowledge exchange mechanism—KEM) about the search region between $s_{1}$ and $s_{2}$ is triggered and it is executed in the following way in every iteration: ifrand(0,1)>kef replace one worst solution from $s_{1}$ ($X_{w,s1}$) with the best individual from $s_{2}$ ($X_{b,s2}$) and vice-versa. However, this mechanism may also render some problems. If the exchange of solutions between swarms is triggered too early and/or too frequently, then diversity of swarms may be lost and local optimal solutions may be returned. This scenario is mitigated by additional two control parameters: ψ and kef. The kef (knowledge exchange frequency) controls the frequency of KEM triggering after the condition t>ψ, where *t* is the current iteration counter, has been satisfied.

High-level inner workings of proposed MS-AFS are described in Algorithm 4.
**Algorithm 4** High-level MS-AFS pseudo-codeInitialize global parameters: t=0, *T*, and *N*.Initialize: control parameters of ABC, FA, and SCA meta-heuristics.Generate initial population *P* according to Algorithm 1.Determine populations for $s_{1}$ and $s_{2}$—$P_{1}$ and $P_{2}$, respectively.**while** t≤T**do**   Execute $s_{1}$ according to Algorithm 2   Execute $s_{2}$ according to Algorithm 3   **if** t>ϕ **then**     **if** rand(0,1)>kef **then**        Trigger KEM mechanism     **end if**   **end if****end while**Return $X_{best}$Results analysis, performance metrics generation and visualization

#### 3.2.3. Computational Complexity, MS-AFS Solutions’ Encoding for ELM Tuning and Flow-Chart

Because the most computationally costly portion of the swarm intelligence algorithm is the objective evaluation [86], the number of FFEs may be used to assess the complexity of the method.

Proposed MS-AFS does not impose additional FFEs, not even in the initialization phases, therefore in terms of FFEs, its complexity is given as:

(22) O(MS−AFS)=O(N)+O((N·T))

However, there is always a trade-off, therefore the proposed MS-AFS also exhibits some limitations. The major drawback of MS-AFS method is reflected in the fact that the algorithm requires more control parameters. All three components of the MS-AFS, namely the ABC, FA, and SCA, have to be tuned with their respective control parameters. Nevertheless, the proposed MS-AFS is significantly more efficient than the individual algorithms, justifying the requirement for more control parameters, as it is shown in Section 4.

The plain ELM model is based on the random initial set of the input weights and biases, consequently being vulnerable to several performance drawbacks. More specifically, the plain ELM frequently requires a significant amount of neurons, that could be not necessary and/or sub-optimal. This increase in the number of neurons in the hidden layer can slow down the ELM response in case that previously unknown data are wired to the network inputs, rendering it impractical for numerous practical applications.

The proposed hybrid multi-swarm meta-heuristics and ELM model framework utilizes MS-AFS meta-heuristics to optimize the input weights and biases of the ELM model, while the number of neurons in the hidden layer was determined by a simple grid search. The MP generalized inverse has been used to obtain the output weights. Therefore, the proposed hybrid technique is named ELM-MS-AFS.

**Figure 1:**
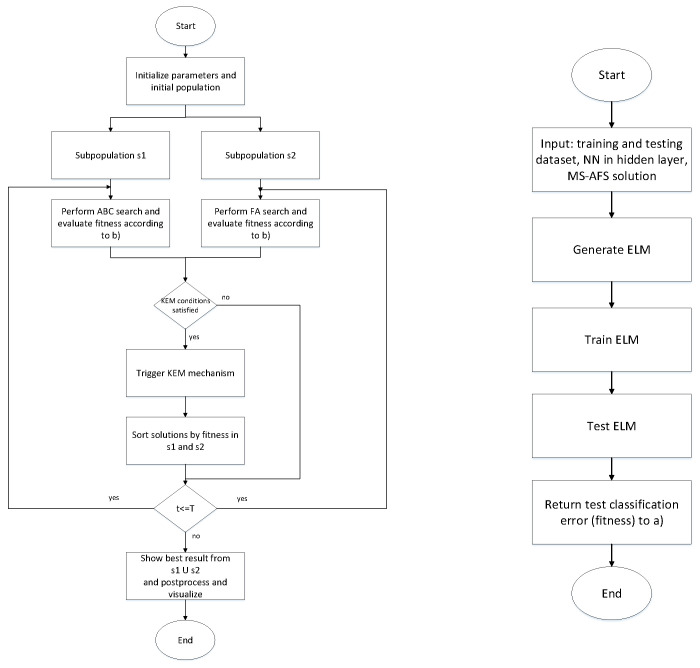
Overview for the proposed ELM-MS-AFS approach.

Each MS-AFS solution consists of nn·fs+nn parameters, where nn and fs denote number of neurons in the hidden layer, and the size of input feature vector, respectively. For the sake of clarity, a flow-chart of proposed ELM-MS-AFS is given in Figure 1.

## 4. Experiments

This section first describes the datasets used in the experiments, followed by the metrics that were used to evaluate the results. Finally, this section provides the obtained results and their comparative analysis with other similar cutting-edge methods.

### 4.1. Datasets

The experiments in this research were performed on seven well-known UCI (University of California, Irvine) benchmark datasets, namely Diabetes, Heart Disease, Iris, Wine, Wine Quality, Satellite and Shuttle, that can be retrieved from https://archive.ics.uci.edu/ml/datasets.php (accessed on 15 May 2022).

Their characteristics have been summarized in Table 1. The Pima Indians Diabetes dataset is utilized in diabetes diagnostics, to determine if the patient is positive or not. The dataset comprises 768 patterns belonging to two distinct classes. The Heart Disease dataset comprises 270 patterns, with 13 attributes and two classes, that indicate if the patient has a heart disease or not. The third dataset, namely the Fisher Iris dataset, consists of three flower species measurements (viz. Setosa, Verginica, and Versicolor). The Iris dataset is comprised of three classes, and every class has fifty samples. The Wine dataset comprises 178 samples belonging to three sorts of wines. The Wine dataset was created by the chemical analyses that have been performed on wines produced from the grapes grown in the same region in Italy, but by three different cultivators.

The fifth dataset used, Wine Quality, deals with the sorts of the Portuguese “Vinho Verde” wines. The quality of wines is modeled by the results obtained with physiochemical testing. The satellite image dataset comprises the multi-spectral pixel values located in 3×3 neighbourhood areas of the satellite images. This dataset is also available on the UCI repository (https://archive.ics.uci.edu/ml/datasets/Statlog+(Landsat+Satellite) (accessed on 15 May 2022)), where it is stated that it has seven classes. However, it actually has just six classes, as reported in Table 1. Finally, the seventh dataset, Shuttle, relates to the radiators’ placement on board of the Space Shuttle, and it comprises 58,000 samples, with nine attributes and separated into seven classes.

All datasets have been divided into training and testing groups. Satellite and Shuttle datasets are available with already predetermined train and test subsets, and they were used accordingly. Diabetes, Disease, Iris, Wine, and Wine Quality datasets do not have predetermined training and testing subsets, as each one of them comes in the form of a singular dataset. Therefore, all five mentioned datasets were subsequently separated into testing and training subsets by utilizing 70% of data for training process, and 30% for testing. Since most of the datasets are imbalanced, data are split in a stratified fashion to maintain the same proportions of class labels in training and testing subsets as in the input dataset.

**Figure 2:**
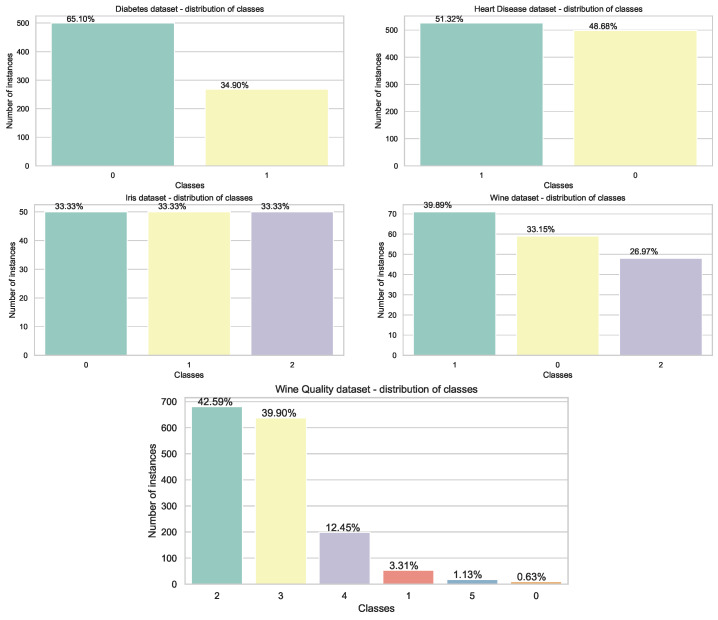
Distribution of classes in Diabetes, Disease, Iris, Wine, and Wine Quality datasets before split.

**Figure 3:**
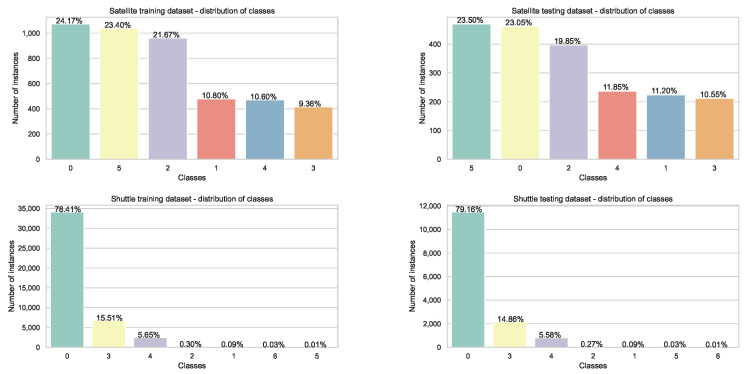
Distribution of classes in Satellite and Shuttle datasets with predetermined training and testing subsets.

Visualization of class distributions in the employed datasets is provided in Figure 2 and Figure 3 for Diabetes, Disease, Iris, Wine, and Wine Quality before split into training and testing subsets and for Satellite and Shuttle with already predetermined training and testing groups, respectively.

### 4.2. Metrics

In order to evaluate the performances of the proposed MS-AFS, it is required to measure them accurately and precisely. The common approach to evaluate machine learning models is based on the false positives (FP) and false negatives (FN), along with true positives (TP) and true negatives (TN), to accurately verify the classification accuracy, as defined by the general formula given by Equation (23).

(23) $$ACC=\left(TP+TN\right)/\left(TP+FP+TN+FN\right)$$

By utilizing TP, TN, FP, and FN, the model’s recall, sensitivity (recall) and F-measure can easily be determined by applying the formulas given in Equations (24)–(26):

(24) Precision=TP/(TP+FP)

(25) Recall(sensitivity)=TP/(TP+FN)

(26) F-measure=(2·Precision·Recall)/(Precision+Recall)

The precision and recall measurements are very important for the imbalanced datasets.

### 4.3. Experimental Results and Comparative Analysis with Other Cutting-Edge Meta-Heuristics

The performance of the suggested method has been evaluated by utilizing the similar experimental setup as proposed in the referred paper [2]. The proposed method has been validated and compared against the basic versions of the algorithms that were used to create a multi-swarm method—ABC [23], FA [24], and SCA [74]. Additionally, the elaborated algorithm has been compared to the bat algorithm (BA) [87], Harris hawk optimization (HHO) [88], whale optimization algorithm (WOA) [27], and Invasive Weed Optimization (IWO) [19], which were also used in [2]. It is important to note that all meta-heuristics included in the experiments were independently implemented by the authors, and these results were reported in the tables. Additionally, to emphasize that meta-heuristics were applied to ELM tuning, each proposed approach is shown with the prefix ‘ELM’.

All meta-heuristics included in comparative analysis were tested with optimal (sub-optimal) parameters which are suggested in original papers. Values of MS-AFS specific parameters were determined empirically and they were set as follows for all simulations: ψ=T/5 and kef=0.6.

In paper [2] simulations were executed with 20 solutions in the population (N=20) and the termination condition was limited to 100 iterations (T=100). However, in this research, a lower number of neurons in the hidden ELM layer were employed for all observed datasets.

In the proposed research, a simple grid search has been applied to determine the optimal (sub-optimal) number of neurons for all datasets in average. The search was performed with 10–200 neurons with a step size of 10 and it was observed that, in average for all datasets, the best performance was obtained with 30, 60, and 90 neurons. Therefore, in this research, simulations with 30, 60, and 90 neurons are conducted to evaluate the performance of the proposed ELM-MS-AFS model.

However, in this research, all methods were tested by employing a substantially lower number of iterations than in [2]. All methods were tested with N=20 and T=20 in 50 independent runs, and best, worst, and mean accuracy along with standard deviation performance metrics are reported in Table 2, Table 3 and Table 4 for 30, 60, and 90 neurons, respectively. The basic ELM was also tested on each dataset in 50 independent runs.

The findings from Table 2, Table 3 and Table 4 demonstrate the superior performance of meta-heuristics-based ELMs over the basic ELM. It can be noted that the plain ELM exhibited high standard deviations on all datasets, for 30, 60, and 90 neurons, which was expected as the weights are initialized in a random fashion, without any kind of “intelligence”. The proposed ELM-MS-AFS approach produced the best results by far, considering the meta-heuristics-based ELMs. In case of 30 neurons in a hidden layer, depicted in Table 2, the ELM-MS-AFS obtained the best results in terms of best, worst, and mean accuracies on five datasets (Diabetes, Disease, Wine Quality, Satellite, and Shuttle), and also being tied on the first place in two occasions (Iris and Wine). Similar trends are observed in case of 60 neurons (Table 3), where ELM-MS-AFS achieved the best results in terms of best, worst, and mean accuracies on three datasets (Diabetes, Disease, Wine Quality, and Satellite), and being tied on the first position on Iris and Wine Datasets. The ELM-MS-AFS also obtained the highest best accuracy in the case of the Shuttle dataset. Finally, on the experiments with 90 neurons in the hidden layer shown in Table 4, the proposed ELM-MS-AFS obtained the best results on five datasets (Diabetes, Disease, Wine Quality, Satellite, and Shuttle), and was tied for the first place on the Wine dataset.

Another interesting conclusion can be derived from the obtained performance for a different amount of neurons in the hidden layer. For example, for the ELM-MS-AFS approach, performance rise with the increased number of neurons on some datasets, as it can be seen for the Disease dataset, where the ELM-MS-AFS achieved an average accuracy of 91.80% with 30 neurons, 92.86% with 60 neurons, and 95.70% with 90 neurons. Similar patterns can be observed for the Satellite dataset. On the other hand, on the Diabetes dataset, ELM-MS-AFS achieved the best performance and average accuracy of 84.63% with 30 neurons, then a drop to 81.60% in the average accuracy can be seen with 60 neurons, and, finally, again an increase to 83.55% with 90 neurons. Finally, for the Wine Quality dataset, the ELM-MS-AFS achieved the best performances with an accuracy of 67.60% with 60 neurons in the hidden layer. A further incrementation in neurons did not result in an increased accuracy, as there is a drop of the average accuracy to 67.40% when the network is leveraged to 90 neurons. This is a classic example of the over-fitting issue, where increasing the number of neurons reduces the generalization capabilities of the model, and results in the network that learns training data too well and under-performs on the test data.

As already noted above, for imbalanced datasets, accuracy metric is not enough to gain insights into classification results, therefore in Table 5, Table 6 and Table 7, macro averaged precision, recall, and f1-score metrics, obtained by ELM tuned with meta-heuristics approaches for the best run, were also shown for experiments with 30, 60, and 90 neurons, respectively. All those metrics were extracted from classification report.

**Figure 4:**
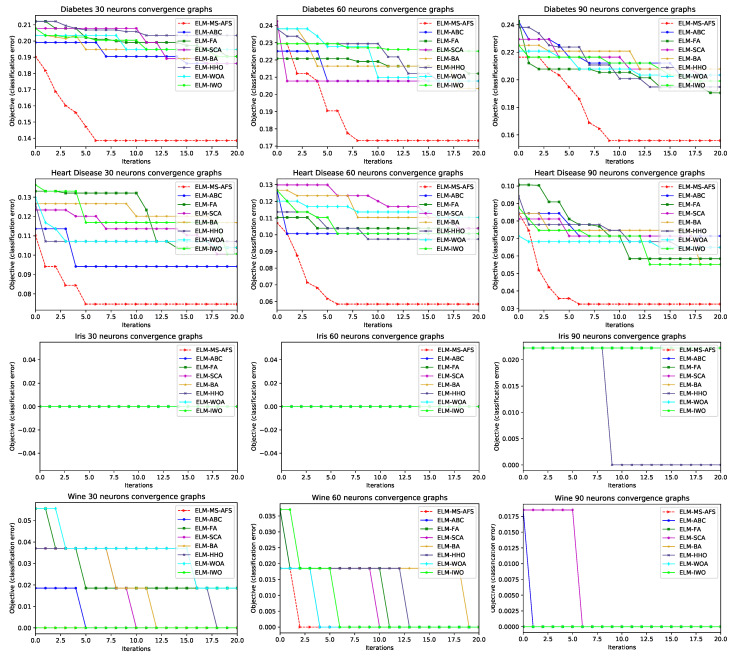

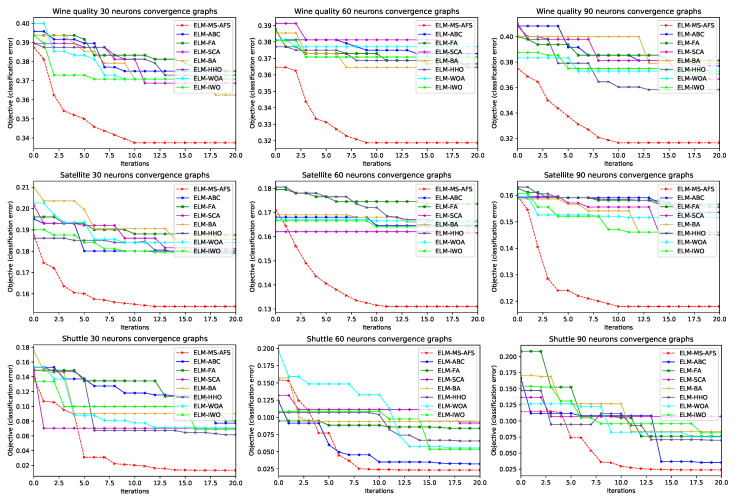
Graphs for convergence speed evaluation on seven observed datasets for 30, 60, and 90 neurons, for the proposed method vs. other approaches.

In order to better visualize the performance and classification error rate speed of convergence for the proposed ELM-MF-AFS method, convergence graphs for all seven datasets, for the cases of 30, 60, and 90 neurons, are shown in Figure 4. The compared algorithms were also plotted in Figure 4. It is obvious that the proposed method converges much faster than other approaches for most of the datasets. Additionally, it can be observed that the proposed MS-AFS has initial advantage due to the chaotic and QRL initialization.

**Figure 5:**
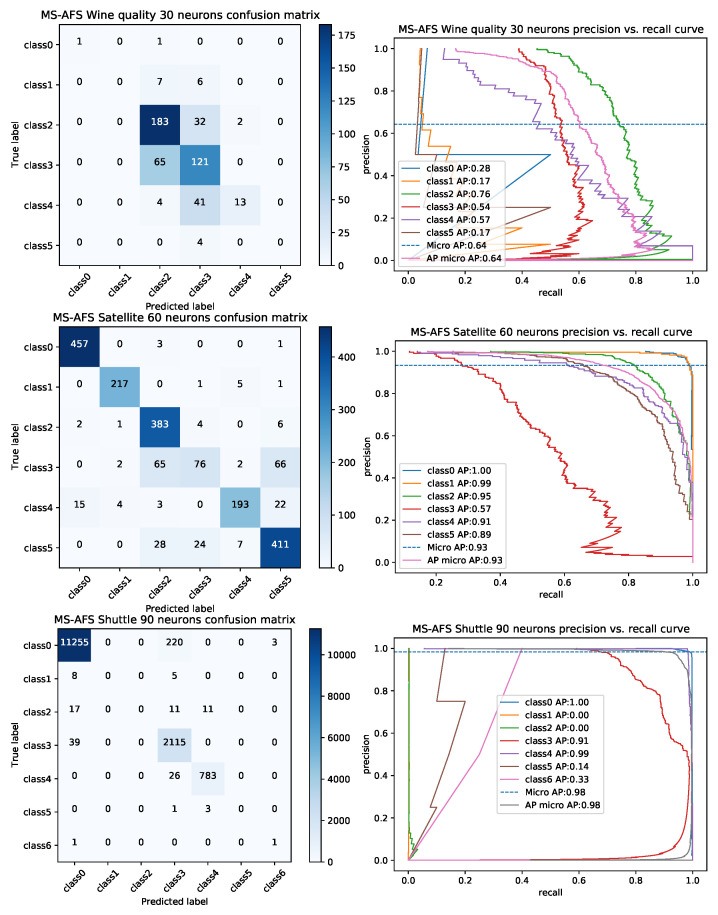
Generated confusion matrices and PR curves for some datasets by ELM-MS-AFS.

Finally, visualization of obtained metrics is further showed in Figure 5, where generated confusion matrices and precision-recall (PR) curves for some simulations by proposed ELM-MS-AFS are shown.

#### Statistical Tests

In this section, findings of statistical tests conducted for simulations shown in Section 4.3, are presented with the goal of establishing whether or not performance improvements of proposed ELM-MS-AFS over other state-of-the-art meta-heuristics are statistically significant.

All statistical tests were performed by taking best values of all methods obtained in all three simulations—with 30, 60, and 90 neurons in the hidden layer. In order to determine if the generated improvements are significant in terms of statistics, a Friedman Aligned test [89,90] and two-way variance analysis by ranks have been employed. By analyzing the test results, a conclusion can be made if there is a significant results’ difference among the proposed ELM-MS-AFS and other methods encompassed by comparison. The Friedman Aligned test results for the eight compared algorithms on seven datasets are presented in Table 8.

The results presented in Table 8 statistically indicate that the proposed ELM-MS-AFS algorithm has superior performance when compared to the other seven algorithms with an average rank value of 9.5. The second best performance was achieved by ELM-HHO algorithm that scored the average rank of 24.36, while the ELM-IWO algorithm obtained the average rank of 27.64 at third place. The basic ELM-ABC, ELM-FA and ELM-SCA meta-heuristics obtained the average ranks of 34.21, 31.07, and 32.5, respectively. Additionally, the Friedman Aligned statistics ($\chi_{r}^{2}=18.49$) is greater than the $\chi^{2}$ critical value with seven degrees of freedom (14.07), at significance level α=0.05. As the result, the null hypothesis ($H_{0}$) can be rejected and it can be stated that the suggested ELM-MS-AFS achieved results that are significantly different than the other seven algorithms.

Finally, the non-parametric post-hoc procedure, the Holm’s step-down procedure, is also conducted and presented in Table 9. By using this procedure, all methods are sorted according to their *p* value and compared with α/(k−i), where *k* and *i* represent the degree of freedom (in this work k=10) and the algorithm number after sorting according to the *p* value in ascending order (which corresponds to rank), respectively. In this study the α is set to 0.05 and 0.1. Additionally, it is noted that the *p*-value results are provided in scientific notation.

The results given in the Table 9 suggest that the proposed algorithm significantly outperformed all opponent algorithms at both significance levels α=0.1 and α=0.05.

### 4.4. Hybridization by Pairs

Although the reasons of combining ABC, FA and SCA meta-heuristics in multi-swarm approach are elaborated in Section 3.2.1, for the purpose of this research, additional methods were implemented to prove that combining two algorithms is not as effective as joining three approaches. Therefore, the following HLH teamwork mode optimizers were implemented: ABC-FA, ABC-SCA, and FA-SCA.

All methods have the same properties as the MS-AFS meta-heuristics—they employ chaotic and QRL population initialization and the KEM procedure controlled by kef and ψ control parameters (for more details please refer to Section 3.2.2). During the initialization phase, N/2 worse individuals are included in population $s_{1}$, which is guided by the ABC algorithm in case of ABC-FA and ABC-SCA approaches, and by the FA algorithm in the case of FA-SCA method. It is also worth mentioning that all three additional hybrid methods have the same computational complexity as the MS-AFS.

It needs to be noted that the hybrid between FA and SCA is established as the HLH, not as the LLH which is the case of the MS-AFS, because only in this way two populations controlled by different methods can be generated. Alternatively, establishing LLH between FA and SCA would not render a fair comparison with the MS-AFS, because the KEM procedure could not be implemented. Naturally, the three methods above can be combined in various different ways, but performing all hybridization possibilities would go far beyond the scope of our research.

The same experimental ELM’s tuning setup as in the basic experiment (Section 4.3) was established and the same control parameters’ values as for ELM-MS-AFS were used for ELM-ABC-FA, ELM-ABC-SCA, and ELM-FA-SCA. The additionally implemented methods were validated only for three more challenging datasets from the previous experiment: Wine Quality, Satellite, and Shuttle with 30, 60, and 90 neurons.

However, with the aim of gaining more insights into the performance of proposed ELM-MS-AFS, one more challenging dataset was included for the current comparison. The newly utilized NSL-KDD dataset is an improved version of the KDD’99 dataset for network intrusion detection and it has been widely used in the modern literature [91,92,93]. However, according to authors’ findings, the ELM has never been applied to this dataset before.

**Figure 6:**
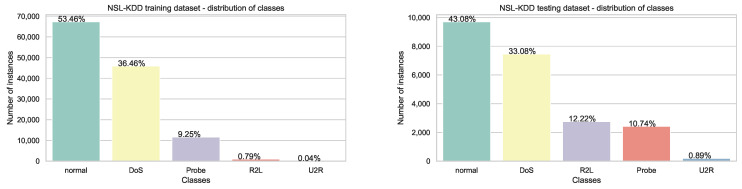
Distribution of classes in NSL-KDD dataset with predetermined training and testing subsets.

Predefined training and testing sets for the NSL-KDD, as well as its description, can be retrieved from the following URL: https://unb.ca/cic/datasets/nsl.html (accessed on 15 May 2022) and it includes in total 148,517 instances with 41 mixed numerical and categorical features along with five classes. Class 0 represents normal network traffic (no intrusion), while the other four classes denote malicious type of network traffic (Probe, DoS, U2R, and R2L). For training ELM, all categorical features were transformed into integers using one hot encoding (OHE) scheme, resulting in a dataset with 122 attributes. Other features are normalized. It also should be emphasized that the NSL-KDD dataset is highly imbalanced (Figure 6) and in the conducted experiments it was used as such.

Following the setup from the previous experiments, all hybrids are tested with N=20 and T=20 in 50 independent runs and best, mean, worst accuracy along with standard deviation for all four datasets with 30, 60, and 90 ELM neurons are captured and reported in Table 10. Detailed performance indicators for the best run in terms of macro averaged precision, recall, and f1-score are shown in Table 11. In both tables, the best achieved results are denoted with bold style.

**Figure 7:**
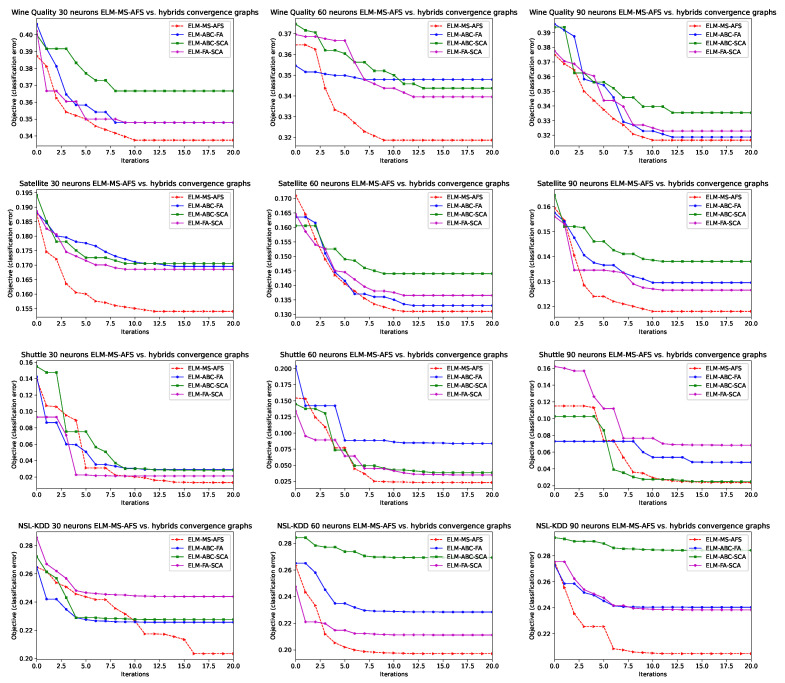
Graphs for convergence speed evaluation on four observed datasets for 30, 60, and 90 neurons, for the proposed ELM-MS-AFS vs. other hybrid approaches.

Convergence speed graphs for all additional simulations are shown in Figure 7.

From provided simulation results, as well as from convergence graphs, it can clearly be stated that the proposed ELM-MS-AFS on average exhibits superior performance over ELM-ABC-FA, ELM-ABC-SCA, and ELM-FA-SCA hybrid meta-heuristics, therefore the assumption that combining three approaches renders better performance than joining two methods is justified. It is also interesting to notice that, on average, when all simulations are taken into account, the ELM-ABC-FA and ELM-FA-SCA are close in terms of performance and that the ELM-ABC-SCA achieves slightly worse results. Finally, by comparing with the metrics established by other state-of-the-art swarm approaches, shown in the tables from Section 4.3, all three hybrid meta-heuristics on average proved to be more efficient and robust optimizers than standard, non-hybridized algorithms.

**Figure 8:**
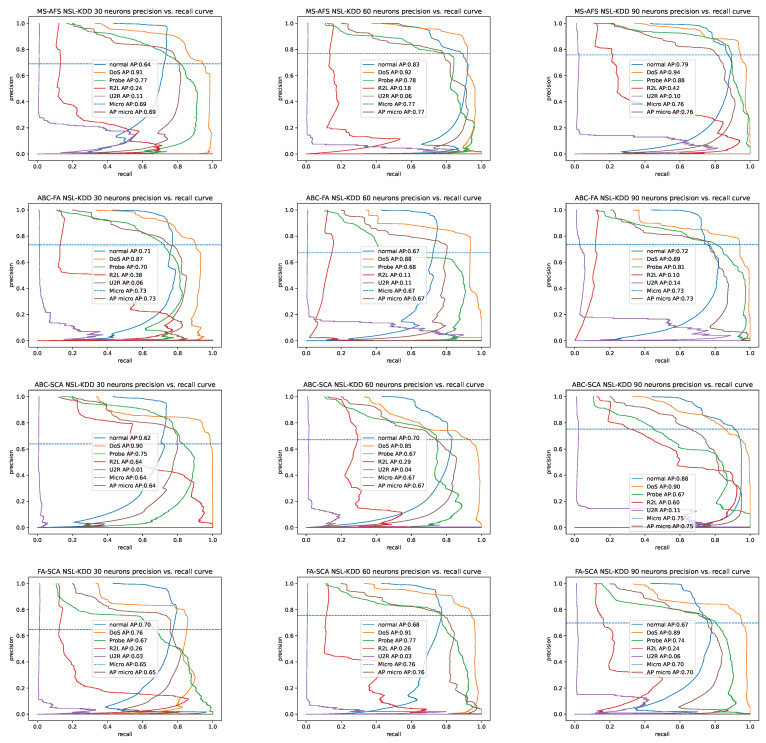
Generated PR curves for NSL-KDD dataset by hybrid methods.

Additionally, since the NSL-KDD dataset is highly imbalanced, the PR curves for all four hybrid methods for simulations with 30, 60, and 90 ELM’s neurons are shown in Figure 8. From this visualization, it can be also concluded that the ELM-MS-AFS on average manages to better classify classes with minority of samples.

## 5. Conclusions

This paper proposes a novel approach to ELM optimization by swarm intelligence meta-heuristics. For this purpose, a novel multi-swarm algorithm has been implemented, by combining three famous algorithms: ABC, FA, and SCA. The goal of this hybrid method was to combine the strengths of each individual algorithm, and compensate their weaknesses. New multi-swarm meta-heuristics has been named MS-AFS, and later used to optimize the weights and biases in ELM model. The number of ELM’s hidden neurons was not subjected to optimization, as the simple grid search was employed to determine the optimal number of neurons.

To validate the new ELM-MS-AFS technique, thorough simulations were conducted with seven UCI benchmark datasets, with 30, 60, and 90 neurons in the hidden layer. The results have been compared to the basic ELM, and to seven other cutting-edge meta-heuristics-based ELMs. The proposed ELM-MS-AFS method has proven to be superior to other methods included in the analysis, as it was confirmed with statistical tests employed to determine the significance of the improvements of the proposed method.

Additionally, to prove that combining two algorithms is not as effective as joining three approaches, hybrids generated by pairing each two methods employed in the proposed multi-swarm approach, were also implemented and validated against four challenging datasets. From obtained simulation results, it was concluded that the proposed ELM-MS-AFS on average exhibits superior performance over ELM-ABC-FA, ELM-ABC-SCA, and ELM-FA-SCA hybrid meta-heuristics, therefore the assumption that combining three approaches renders better performance than joining two methods is justified.

The future research in this area will include extensive testing of the proposed ELM-MS-AFS approach on other benchmark and real-life datasets, and employing it in various application domains. Additionally, the number of neurons in the hidden layer will also be subjected to the optimization process. Finally, the proposed MS-AFS meta-heuristics will be tested and employed for solving NP-hard tasks for other domains, such as wireless sensor networks and cloud-based systems.

## Figures and Tables

**Table 1:** Datasets used in the conducted experiments.

Dataset	Samples	Training Data	Testing Data	Attributes	Classes
Diabetes	768	538	230	8	2
Disease	270	189	81	13	2
Iris	150	105	45	4	3
Wine	178	125	53	13	3
Wine Quality	1599	1120	479	11	6
Satellite	6400	5400	1000	36	6
Shuttle	58,000	50,750	7250	9	7

**Table 2:** Accuracy comparative analysis for simulations with 30 neurons.

		Diabetes	Disease	Iris	Wine	Wine Quality	Satellite	Shuttle
ELM	best (%)	73.59	87.99	80	98.15	60	77.24	84.12
	worst (%)	61.90	79.87	66.67	83.33	54.37	68.48	10.39
	mean (%)	71.22	84.67	72.18	92.52	57	73.68	55.44
	std	2.5628	1.9972	3.9811	3.7214	1.2527	2.3860	24.6222
ELM-IWO	best (%)	80.95	89.94	100.00	100.00	62.92	82.04	93.13
	worst (%)	80.09	88.96	100.00	100.00	62.29	81.59	91.14
	mean (%)	80.52	89.61	100.00	100.00	62.76	81.79	92.20
	std	0.0061	0.0046	0.0000	0.0000	0.0031	0.0023	0.0100
ELM-WOA	best (%)	80.52	89.61	100.00	98.15	62.92	81.59	92.90
	worst (%)	79.22	87.01	100.00	96.30	61.67	80.34	88.43
	mean (%)	79.87	88.72	100.00	97.69	62.29	80.94	90.93
	std	0.0092	0.0123	0.0000	0.0093	0.0051	0.0067	0.0228
ELM-HHO	best (%)	79.65	89.29	100.00	100.00	62.71	82.09	93.85
	worst (%)	79.22	87.34	100.00	94.44	61.25	80.89	88.08
	mean (%)	79.44	88.56	100.00	98.15	61.82	81.44	91.24
	std	0.0031	0.0093	0.0000	0.0262	0.0071	0.0062	0.0292
ELM-BA	best (%)	80.52	88.31	100.00	100.00	63.75	81.44	90.98
	worst (%)	80.09	87.66	100.00	100.00	61.67	80.64	89.86
	mean (%)	80.30	88.07	100.00	100.00	62.66	81.09	90.39
	std	0.0031	**0.0031**	0.0000	0.0000	0.0086	0.0039	0.0056
ELM-SCA	best (%)	81.39	89.94	100.00	100.00	63.13	81.89	92.97
	worst (%)	80.95	88.31	100.00	100.00	61.67	81.49	89.75
	mean (%)	81.17	89.12	100.00	100.00	62.50	81.72	91.83
	std	0.0031	0.0077	0.0000	0.0000	0.0066	**0.0017**	0.018
ELM-FA	best (%)	80.95	89.61	100.00	98.15	62.50	81.24	91.97
	worst (%)	80.95	87.66	100.00	96.30	61.46	80.54	89.95
	mean (%)	80.95	88.72	100.00	97.22	61.98	80.90	91.11
	std	**0.0000**	0.0081	0.0000	0.0107	0.0043	0.0032	0.0104
ELM-ABC	best (%)	81.39	90.58	100.00	100.00	62.50	81.99	92.28
	worst (%)	80.09	88.64	100.00	100.00	60.21	81.29	88.82
	mean (%)	80.74	89.37	100.00	100.00	61.72	81.73	90.65
	std	0.0092	0.0093	0.0000	0.0000	0.0104	0.0032	0.0174
ELM-MS-AFS	best (%)	**86.15**	**92.53**	100.00	100.00	**66.25**	**84.59**	**98.67**
	worst (%)	**83.12**	**90.91**	100.00	100.00	**65.63**	**82.99**	**97.71**
	mean (%)	**84.63**	**91.80**	100.00	100.00	**65.99**	**83.67**	**98.21**
	std	0.0214	0.0072	0.0000	0.0000	**0.0026**	0.0076	**0.0048**

**Table 3:** Accuracy comparative analysis for simulations with 60 neurons.

		Diabetes	Disease	Iris	Wine	Wine Quality	Satellite	Shuttle
ELM	best (%)	69.69	89.93	71.11	94.44	58.96	80.09	90.00
	worst (%)	55.84	83.12	40	81.48	52.29	74.59	3.04
	mean (%)	64.85	86.52	54.4	89.48	55.87	78.11	42.43
	std	3.9713	1.4753	7.9534	3.9447	1.9326	1.2749	32.6834
ELM-IWO	best (%)	77.49	89.94	100.00	100.00	62.92	83.59	94.64
	worst (%)	77.49	88.96	100.00	100.00	61.67	83.34	88.22
	mean (%)	77.49	89.45	100.00	100.00	62.24	83.47	91.42
	std	0.0107	0.0042	0.0000	0.0000	0.0057	0.0018	0.0321
ELM-WOA	best (%)	79.22	88.96	100.00	100.00	62.29	83.34	94.45
	worst (%)	77.49	88.31	100.00	98.15	61.88	82.64	**91.52**
	mean (%)	78.35	88.64	100.00	99.07	62.08	82.99	**92.67**
	std	0.0122	0.0027	0.0000	0.0107	**0.0017**	0.0050	0.0156
ELM-HHO	best (%)	79.22	90.26	100.00	100.00	63.54	83.34	93.43
	worst (%)	77.92	87.99	100.00	98.15	62.08	83.34	89.32
	mean (%)	78.57	89.29	100.00	99.07	63.07	83.34	91.90
	std	0.0092	0.0103	0.0000	0.0107	0.0069	**0.0000**	0.0224
ELM-BA	best (%)	79.65	88.96	100.00	100.00	63.54	83.39	90.61
	worst (%)	78.35	88.31	97.78	98.15	62.29	82.84	87.46
	mean (%)	79.00	88.56	97.78	99.54	62.76	83.12	88.79
	std	0.0092	0.0031	0.0000	0.0093	0.0060	0.0039	0.0163
ELM-SCA	best (%)	79.22	89.61	100.00	100.00	62.92	83.84	90.83
	worst (%)	77.49	88.64	100.00	98.15	61.25	83.54	89.46
	mean (%)	78.35	89.29	100.00	99.54	62.19	83.69	90.10
	std	0.0122	0.0046	0.0000	0.0093	0.0086	0.0021	**0.0069**
ELM-FA	best (%)	78.79	89.61	100.00	100.00	63.33	82.64	91.59
	worst (%)	77.49	88.64	100.00	100.00	62.08	82.54	85.79
	mean (%)	78.14	89.29	100.00	100.00	62.45	82.59	88.67
	std	0.0092	0.0046	0.0000	0.0000	0.0060	0.0007	0.0290
ELM-ABC	best (%)	79.22	89.94	100.00	100.00	62.71	83.54	96.77
	worst (%)	79.22	89.29	100.00	100.00	62.29	83.34	87.72
	mean (%)	79.22	89.61	100.00	100.00	62.55	83.44	91.83
	std	**0.0000**	0.0027	0.0000	0.0000	0.0002	0.0014	0.0458
ELM-MS-AFS	best (%)	**82.68**	**94.16**	100.00	100.00	**68.13**	**86.89**	**97.68**
	worst (%)	**80.52**	**91.88**	100.00	100.00	**66.88**	**85.89**	84.74
	mean (%)	**81.60**	**92.86**	100.00	100.00	**67.60**	**86.39**	91.62
	std	0.0153	0.0103	0.0000	0.0000	0.0052	0.0071	0.0651

**Table 4:** Accuracy comparative analysis for simulations with 90 neurons.

		Diabetes	Disease	Iris	Wine	Wine Quality	Satellite	Shuttle
ELM	best (%)	70.56	92.53	75.55	90.74	61.04	80.64	80.42
	worst (%)	61.04	83.44	57.78	40.74	46.04	77.59	4.70
	mean (%)	65.56	87.83	67.29	71.48	53.79	79.43	44.12
	std	2.2178	2.1791	4.5550	10.5018	3.1374	0.7149	29.1458
ELM-IWO	best (%)	80.09	94.48	97.78	100.00	62.92	85.39	91.84
	worst (%)	79.65	92.86	97.78	100.00	61.25	84.84	89.19
	mean (%)	79.87	93.34	97.78	100.00	62.24	85.12	90.29
	std	0.0030	0.0077	0.0000	0.0000	0.0071	0.0039	0.0138
ELM-WOA	best (%)	79.65	93.51	97.78	100.00	62.71	84.84	92.49
	worst (%)	79.22	92.53	97.78	100.00	60.21	83.94	89.05
	mean (%)	79.44	93.18	97.78	100.00	61.61	84.39	90.27
	std	0.0031	0.0046	0.0000	0.0000	0.0112	0.0064	0.0193
ELM-HHO	best (%)	80.52	93.51	100.00	100.00	64.17	84.34	93.00
	worst (%)	77.22	92.53	97.78	100.00	61.25	84.04	84.74
	mean (%)	79.87	92.86	98.33	100.00	61.60	84.19	87.72
	std	0.0092	0.0046	0.0111	0.0000	0.0121	0.0021	0.0458
ELM-BA	best (%)	79.22	94.48	97.78	100.00	62.08	85.39	91.63
	worst (%)	78.35	88.31	97.78	100.00	61.67	82.84	88.12
	mean (%)	79.00	88.56	97.78	100.00	62.66	83.12	90.29
	std	0.0092	0.0031	0.0000	0.0000	0.0086	0.0039	0.0189
ELM-SCA	best (%)	79.65	93.51	97.78	100.00	63.33	85.49	89.29
	worst (%)	79.65	92.53	97.78	100.00	61.88	84.59	85.52
	mean (%)	79.65	93.02	97.78	100.00	62.34	85.04	86.87
	std	**0.0000**	0.0042	0.0000	0.0000	0.0069	0.0063	0.0210
ELM-FA	best (%)	80.95	94.16	100.00	100.00	61.88	84.44	92.39
	worst (%)	79.65	92.86	97.78	100.00	60.42	82.54	90.99
	mean (%)	80.30	93.59	98.33	100.00	61.09	82.59	91.70
	std	0.0092	0.0067	0.0111	0.0000	0.0069	**0.0007**	**0.0070**
ELM-ABC	best (%)	79.65	92.86	97.78	100.00	62.29	84.64	96.47
	worst (%)	78.35	89.29	97.78	100.00	61.04	83.34	89.68
	mean (%)	79.00	89.61	97.78	100.00	61.56	83.44	92.61
	std	0.0092	**0.0026**	0.0000	0.0000	**0.0055**	0.0014	0.0349
ELM-MS-AFS	best (%)	**84.42**	**96.75**	97.78	100.00	**68.33**	**88.19**	**97.62**
	worst (%)	**82.68**	**95.13**	97.78	100.00	**66.25**	**87.74**	**93.13**
	mean (%)	**83.55**	**95.70**	97.78	100.00	**67.40**	**87.97**	**94.70**
	std	0.0122	0.0072	0.0000	0.0000	0.0100	0.0072	0.0253

**Table 5:** Precision, recall, and f1-score comparative analysis for simulations with 30 neurons.

		Diabetes	Disease	Iris	Wine	Wine Quality	Satellite	Shuttle
ELM-IWO	accuracy (%)	80.95	89.94	100.00	100.00	62.92	82.04	93.13
	precision	0.789	0.900	1.000	1.000	0.294	0.806	0.391
	recall	0.754	0.899	1.000	1.000	0.287	0.765	0.338
	f1-score	0.767	0.899	1.000	1.000	0.286	0.769	0.358
ELM-WOA	accuracy (%)	80.52	89.61	100.00	98.15	62.92	81.59	92.90
	precision	0.784	0.898	1.000	0.978	0.344	0.708	0.335
	recall	0.747	0.895	1.000	0.984	0.267	0.744	0.313
	f1-score	0.760	0.896	1.000	0.980	0.267	0.719	0.312
ELM-HHO	accuracy (%)	79.65	89.29	100.00	100.00	62.71	82.09	93.85
	precision	0.777	0.894	1.000	1.000	0.303	0.827	0.397
	recall	0.730	0.892	1.000	1.000	0.274	0.755	0.382
	f1-score	0.745	0.893	1.0	1.000	0.274	0.735	0.387
ELM-BA	accuracy (%)	80.52	88.31	100.00	100.00	63.75	81.44	90.98
	precision	0.800	0.887	1.000	1.000	0.318	0.866	0.244
	recall	0.729	0.882	1.000	1.000	0.272	0.744	0.351
	f1-score	0.748	0.883	1.000	1.000	0.271	0.718	0.271
ELM-SCA	accuracy (%)	81.39	89.94	100.00	100.00	63.13	81.89	92.97
	precision	0.818	0.900	1.000	1.000	0.310	**0.868**	0.342
	recall	0.735	0.899	1.000	1.000	0.270	0.752	0.381
	f1-score	0.757	0.899	1.000	1.000	0.270	0.724	0.352
ELM-FA	accuracy (%)	80.95	89.61	100.00	98.15	62.50	81.24	91.97
	precision	0.797	0.899	1.0	0.985	0.313	0.699	0.407
	recall	0.743	0.895	1.000	0.982	0.272	0.748	0.356
	f1-score	0.760	0.896	1.000	0.983	0.275	0.718	0.372
ELM-ABC	accuracy (%)	81.39	90.58	100.00	100.00	62.50	81.99	92.28
	precision	0.805	0.906	1.000	1.000	0.372	0.205	0.347
	recall	0.746	0.906	1.000	1.000	0.285	0.167	0.343
	f1-score	0.764	0.906	1.000	1.000	0.297	0.065	0.345
ELM-MS-AFS	accuracy (%)	**86.15**	**92.53**	100.00	100.00	**66.25**	**84.59**	**98.67**
	precision	**0.857**	**0.926**	1.000	1.000	**0.527**	0.841	**0.564**
	recall	**0.814**	**0.925**	1.000	1.000	**0.370**	**0.793**	**0.436**
	f1-score	**0.830**	**0.925**	1.000	1.000	**0.402**	**0.792**	**0.448**

**Table 6:** Precision, recall, and f1-score comparative analysis for simulations with 60 neurons.

		Diabetes	Disease	Iris	Wine	Wine Quality	Satellite	Shuttle
ELM-IWO	accuracy (%)	77.49	89.94	100.00	100.00	62.92	83.59	94.64
	precision	0.747	0.899	1.000	1.000	0.285	0.826	**0.538**
	recall	0.750	0.900	1.000	1.000	0.296	0.778	**0.422**
	f1-score	0.748	0.899	1.000	1.000	0.290	0.747	**0.427**
ELM-WOA	accuracy (%)	79.22	88.96	100.00	100.00	62.29	83.34	94.45
	precision	0.768	0.890	1.000	1.000	0.277	**0.885**	0.377
	recall	0.756	0.889	1.000	1.000	0.253	0.770	0.384
	f1-score	0.761	0.889	1.000	1.000	0.239	0.749	0.380
ELM-HHO	accuracy (%)	79.22	90.26	100.00	100.00	63.54	83.34	93.43
	precision	0.769	0.902	1.000	1.000	0.277	0.850	0.366
	recall	0.753	0.902	1.000	1.000	0.271	0.771	0.374
	f1-score	0.760	0.902	1.000	1.000	0.265	0.749	0.370
ELM-BA	accuracy (%)	79.65	88.96	100.00	100.00	63.54	83.39	90.61
	precision	0.773	0.890	1.000	1.000	0.304	0.846	0.231
	recall	0.760	0.889	1.000	1.000	0.297	0.772	0.260
	f1-score	0.766	0.889	1.000	1.000	0.296	0.748	0.244
ELM-SCA	accuracy (%)	79.22	89.61	100.00	100.00	62.92	83.84	90.83
	precision	0.767	0.896	1.000	1.000	0.285	0.831	0.400
	recall	0.763	0.896	1.000	1.000	0.296	0.787	0.325
	f1-score	0.765	0.896	1.000	1.000	0.290	0.781	0.353
ELM-FA	accuracy (%)	78.79	89.61	100.00	100.00	63.33	82.64	91.59
	precision	0.768	0.898	1.000	1.000	0.320	0.794	0.353
	recall	0.737	0.895	1.000	1.000	0.282	0.759	0.311
	f1-score	0.748	0.896	1.000	1.000	0.284	0.742	0.308
ELM-ABC	accuracy (%)	79.22	89.94	100.00	100.00	62.71	83.54	96.77
	precision	0.770	0.900	1.000	1.000	0.263	0.815	0.410
	recall	0.750	0.898	1.000	1.000	0.257	0.778	0.410
	f1-score	0.758	0.899	1.000	1.000	0.246	0.756	0.410
ELM-MS-AFS	accuracy (%)	**82.68**	**94.16**	100.00	100.00	**68.13**	**86.89**	**97.68**
	precision	**0.805**	**0.942**	1.000	1.000	**0.550**	0.866	0.412
	recall	**0.805**	**0.942**	1.000	1.000	**0.357**	**0.829**	0.420
	f1-score	**0.805**	**0.942**	1.000	1.000	**0.386**	**0.835**	0.416

**Table 7:** Precision, recall and f1-score comparative analysis for simulations with 90 neurons.

		Diabetes	Disease	Iris	Wine	Wine Quality	Satellite	Shuttle
ELM-IWO	accuracy (%)	80.09	94.48	97.78	100.00	62.92	85.39	91.84
	precision	0.789	0.945	0.978	1.000	0.335	0.850	0.404
	recall	0.772	0.945	0.980	1.000	0.290	0.808	0.357
	f1-score	0.779	0.945	0.978	1.000	0.294	0.814	0.371
ELM-WOA	accuracy (%)	79.65	93.51	97.78	100.00	62.71	84.84	92.49
	precision	0.780	0.935	0.978	1.000	**0.627**	0.838	0.239
	recall	0.781	0.935	0.980	1.000	**0.627**	0.792	0.273
	f1-score	0.781	0.935	0.978	1.000	**0.627**	0.780	0.254
ELM-HHO	accuracy (%)	80.52	93.51	100.00	100.00	64.17	84.34	93.00
	precision	0.790	0.935	1.000	1.000	0.315	**0.889**	0.409
	recall	0.788	0.935	1.000	1.000	0.289	0.782	0.367
	f1-score	0.789	0.935	1.000	1.000	0.290	0.751	0.380
ELM-BA	accuracy (%)	79.22	94.48	97.78	100.00	62.08	85.39	91.63
	precision	0.776	0.945	0.978	1.000	0.325	0.840	0.370
	recall	0.786	0.944	0.980	1.000	0.288	0.818	0.317
	f1-score	0.780	0.945	0.978	1.000	0.291	0.823	0.312
ELM-SCA	accuracy (%)	79.65	93.51	97.78	100.00	63.33	85.49	89.29
	precision	0.780	0.935	0.978	1.000	0.356	0.841	0.321
	recall	0.779	0.935	0.980	1.000	0.305	0.822	0.316
	f1-score	0.780	0.935	0.978	1.000	0.313	0.826	0.309
ELM-FA	accuracy (%)	80.95	94.16	100.00	100.00	61.88	84.44	92.39
	precision	0.796	0.941	1.000	1.000	0.319	0.833	0.412
	recall	0.786	0.942	1.000	1.000	0.268	0.788	0.360
	f1-score	0.791	0.941	1.000	1.000	0.267	0.784	0.375
ELM-ABC	accuracy (%)	79.65	92.86	95.56	100.00	62.29	84.64	96.47
	precision	0.796	0.930	0.958	1.000	0.330	0.830	0.402
	recall	0.753	0.929	0.956	1.000	0.284	0.798	0.384
	f1-score	0.765	0.928	0.955	1.000	0.288	0.799	0.391
ELM-MS-AFS	accuracy (%)	**84.42**	**96.75**	97.78	100.00	**68.33**	**88.19**	**97.62**
	precision	**0.844**	**0.967**	0.978	1.000	0.345	0.878	**0.445**
	recall	**0.814**	**0.968**	0.980	1.000	0.321	**0.853**	**0.490**
	f1-score	**0.825**	**0.967**	0.978	1.000	0.325	**0.860**	**0.461**

**Table 8:** Friedman Aligned test ranks for the compared algorithms.

Dataset	ELM-ABC	ELM-FA	ELM-SCA	ELM-BA	ELM-HHO	ELM-WOA	ELM-IWO	ELM-MS-AFS
Diabetes	45	12	45	53	26	45	34	2
Disease	51	21	38	10.5	38	38	10.5	6
Iris	30.5	30.5	30.5	30.5	7	30.5	30.5	9
Wine	16.5	16.5	16.5	16.5	16.5	16.5	16.5	16.5
Wine Quality	49	55	27	52	8	42	35	1
Satellite	43	47	22	24.5	48	40	24.5	4
Shuttle	5	41	56	54	23	36	50	3
Average	34.28	31.86	33.57	34.43	23.78	35.43	28.71	5.93
Rank	6	4	5	7	2	8	3	1

**Table 9:** Results of the Holm’s step-down procedure.

Comparison	*p*-Value	Rank	0.05/(k−i)	0.1/(k−i)
MS-AFS vs. ABC	$1.92\times 10^{-3}$	0	0.007143	0.014286
MS-AFS vs. BA	$1.92\times 10^{-3}$	1	0.008333	0.016667
MS-AFS vs. FA	$3.76\times 10^{-3}$	2	0.01	0.02
MS-AFS vs. WOA	$3.76\times 10^{-3}$	3	0.0125	0.025
MS-AFS vs. SCA	$5.17\times 10^{-3}$	4	0.016667	0.033333
MS-AFS vs. IWO	$2.17\times 10^{-2}$	5	0.025	0.05
MS-AFS vs. HHO	$4.04\times 10^{-2}$	6	0.05	0.1

**Table 10:** Accuracy comparative analysis—ELM-MS-AFS vs. hybrids.

		Wine Quality	Satellite	Shuttle	NSL-KDD
Results for ELM with 30 neurons					
ELM-ABC-FA	best (%)	65.21	83.04	97.08	77.43
	worst (%)	63.54	82.69	92.96	73.96
	mean (%)	64.17	82.87	95.32	75.26
	std	0.0091	**0.0018**	0.0213	0.0189
ELM-ABC-SCA	best (%)	63.33	82.94	97.17	77.24
	worst (%)	62.29	82.19	84.72	72.90
	mean (%)	62.85	82.51	91.09	75.41
	std	0.0052	0.0039	0.0623	0.0225
ELM-FA-SCA	best (%)	65.21	83.14	97.88	75.60
	worst (%)	62.50	82.52	96.81	75.14
	mean (%)	63.61	82.91	97.40	75.45
	std	0.0142	0.0032	0.0054	**0.0027**
ELM-MS-AFS	best (%)	**66.25**	**84.59**	**98.67**	**79.66**
	worst (%)	**65.63**	**82.99**	**97.71**	**76.59**
	mean (%)	**65.99**	**83.67**	**98.21**	**77.74**
	std	**0.0026**	0.0076	**0.0048**	0.0167
Results for ELM with 60 neurons					
ELM-ABC-FA	best (%)	65.21	86.69	91.62	77.16
	worst (%)	60.83	84.69	85.57	74.53
	mean (%)	63.65	85.54	89.53	75.72
	std	0.0194	0.0103	0.0343	0.0133
ELM-ABC-SCA	best (%)	65.63	85.59	96.15	73.07
	worst (%)	62.08	84.54	**92.15**	71.77
	mean (%)	63.54	84.94	93.88	72.35
	std	0.0160	**0.0057**	**0.0205**	**0.0066**
ELM-FA-SCA	best (%)	66.04	86.34	96.51	78.88
	worst (%)	61.67	84.84	91.70	75.11
	mean (%)	64.22	85.83	**93.97**	76.84
	std	0.0190	0.0085	0.0241	0.0190
ELM-MS-AFS	best (%)	**68.13**	**86.89**	**97.68**	**80.29**
	worst (%)	**66.88**	**85.89**	84.74	**75.55**
	mean (%)	**67.60**	**86.39**	91.62	**78.42**
	std	**0.0052**	0.0071	0.0651	0.0252
Results for ELM with 90 neurons					
ELM-ABC-FA	best (%)	68.13	87.04	95.21	75.96
	worst (%)	63.96	85.19	90.96	73.59
	mean (%)	66.30	86.03	92.80	74.95
	std	0.0173	0.0094	**0.0218**	0.0122
ELM-ABC-SCA	best (%)	66.46	86.19	97.52	71.58
	worst (%)	64.38	85.44	90.66	69.47
	mean (%)	65.57	85.78	**95.01**	70.87
	std	0.0087	0.0038	0.0378	0.0122
ELM-FA-SCA	best (%)	67.71	87.34	93.17	76.16
	worst (%)	**66.46**	87.19	83.27	74.68
	mean (%)	67.03	87.26	84.73	75.62
	std	**0.0052**	**0.0008**	0.0535	**0.0082**
ELM-MS-AFS	best (%)	**68.33**	**88.19**	**97.62**	**79.52**
	worst (%)	66.25	**87.74**	**93.13**	**75.34**
	mean (%)	**67.40**	**87.97**	94.70	**77.43**
	std	0.0100	0.0072	0.0253	0.0209

**Table 11:** Precision, recall, and f1-score comparative analysis—ELM-MS-AFS vs. hybrids.

		Wine Quality	Satellite	Shuttle	NSL-KDD
Results for ELM with 30 neurons					
ELM-ABC-FA	accuracy (%)	65.21	83.04	97.08	77.43
	precision (%)	0.327	0.819	0.408	0.473
	recall (%)	0.326	0.764	0.420	0.483
	f1-score	0.325	0.740	0.413	0.470
ELM-ABC-SCA	accuracy (%)	63.33	82.94	97.17	77.24
	precision (%)	0.320	0.873	0.404	0.470
	recall (%)	0.319	0.765	0.417	0.511
	f1-score	0.318	0.735	0.408	0.491
ELM-FA-SCA	accuracy (%)	65.21	83.14	97.88	75.60
	precision (%)	0.328	**0.882**	0.413	0.453
	recall (%)	0.324	0.768	0.422	0.490
	f1-score	0.324	0.739	0.417	0.468
ELM-MS-AFS	accuracy (%)	**66.25**	**84.59**	**98.67**	**79.66**
	precision (%)	**0.527**	0.841	**0.564**	**0.492**
	recall (%)	**0.370**	**0.793**	**0.436**	**0.518**
	f1-score	**0.402**	**0.792**	**0.448**	**0.500**
Results for ELM with 60 neurons					
ELM-ABC-FA	accuracy (%)	65.21	86.69	91.62	77.16
	precision (%)	0.328	0.863	**0.537**	0.485
	recall (%)	0.305	**0.835**	0.357	0.499
	f1-score	0.306	**0.841**	0.406	0.486
ELM-ABC-SCA	accuracy (%)	65.63	85.59	96.15	73.07
	precision (%)	0.405	0.853	0.403	0.461
	recall (%)	0.305	0.808	0.414	0.477
	f1-score	0.311	0.804	0.408	0.460
ELM-FA-SCA	accuracy (%)	66.04	86.34	96.51	78.88
	precision (%)	0.495	0.855	0.412	0.485
	recall (%)	0.311	0.825	0.383	0.521
	f1-score	0.320	0.831	0.395	0.499
ELM-MS-AFS	accuracy (%)	**68.13**	**86.89**	**97.68**	**80.29**
	precision (%)	**0.550**	**0.866**	0.412	**0.486**
	recall (%)	**0.357**	0.829	**0.420**	**0.540**
	f1-score	**0.386**	0.835	**0.416**	**0.511**
Results for ELM with 90 neurons					
ELM-ABC-FA	accuracy (%)	68.13	87.04	95.21	75.96
	precision (%)	0.377	0.861	0.389	0.495
	recall (%)	0.342	0.834	0.370	0.498
	f1-score	0.345	0.838	0.376	0.488
ELM-ABC-SCA	accuracy (%)	66.46	86.19	97.52	71.58
	precision (%)	0.370	**0.879**	0.411	0.475
	recall (%)	0.340	0.810	0.418	0.452
	f1-score	0.347	0.807	0.414	0.449
ELM-FA-SCA	accuracy (%)	67.71	87.34	93.17	76.16
	precision (%)	**0.486**	0.865	0.415	0.495
	recall (%)	**0.486**	0.839	0.367	0.495
	f1-score	**0.453**	0.845	0.383	0.486
ELM-MS-AFS	accuracy (%)	**68.33**	**88.19**	**97.62**	**79.52**
	precision (%)	0.345	0.878	**0.445**	**0.512**
	recall (%)	0.321	**0.853**	**0.490**	**0.525**
	f1-score	0.325	**0.860**	**0.461**	**0.513**

## Data Availability

All datasets used in this study are public and available on the UCI repository on the following URL: https://archive.ics.uci.edu/ml/datasets.php, accessed on 15 May 2022. Preprocessed datasets along with same code is available on the following GitHub link: https://github.com/nbacanin/sensorsELM2022, accessed on 15 May 2022.

## References

[1] Huang G.B., Zhu Q.Y., Siew C.K. Extreme learning machine: A new learning scheme of feedforward neural networks. Proceedings of the 2004 IEEE International Joint Conference on Neural Networks (IEEE Cat. No.04CH37541).

[2] Alshamiri A.K., Singh A., Surampudi B.R. (2018). Two swarm intelligence approaches for tuning extreme learning machine. Int. J. Mach. Learn. Cybern..

[3] Wang J., Lu S., Wang S., Zhang Y.D. (2021). A review on extreme learning machine. Multimed. Tools Appl..

[4] Rong H.J., Ong Y.S., Tan A.H., Zhu Z. (2008). A fast pruned-extreme learning machine for classification problem. Neurocomputing.

[5] Zhu Q.Y., Qin A., Suganthan P., Huang G.B. (2005). Evolutionary extreme learning machine. Pattern Recognit..

[6] Cao J., Lin Z., Huang G.B. (2012). Self-adaptive evolutionary extreme learning machine. Neural Process. Lett..

[7] Miche Y., Sorjamaa A., Bas P., Simula O., Jutten C., Lendasse A. (2009). OP-ELM: Optimally pruned extreme learning machine. IEEE Trans. Neural Netw..

[8] Huang G.B., Chen L., Siew C.K. (2006). Universal approximation using incremental constructive feedforward networks with random hidden nodes. IEEE Trans. Neural Netw..

[9] Serre D. (2002). Matrices: Theory and Applications.

[10] Huang G.B., Zhu Q.Y., Siew C.K. (2006). Extreme learning machine: Theory and applications. Neurocomputing.

[11] Huang G.B. (2003). Learning capability and storage capacity of two-hidden-layer feedforward networks. IEEE Trans. Neural Netw..

[12] Zheng W., Qian Y., Lu H. (2013). Text categorization based on regularization extreme learning machine. Neural Comput. Appl..

[13] Zong W., Huang G.B. (2011). Face recognition based on extreme learning machine. Neurocomputing.

[14] Cao F., Liu B., Park D.S. (2013). Image classification based on effective extreme learning machine. Neurocomputing.

[15] Wang Z., Yu G., Kang Y., Zhao Y., Qu Q. (2014). Breast tumor detection in digital mammography based on extreme learning machine. Neurocomputing.

[16] Kaya Y., Uyar M. (2013). A hybrid decision support system based on rough set and extreme learning machine for diagnosis of hepatitis disease. Appl. Soft Comput..

[17] Xu Y., Shu Y. (2006). Evolutionary extreme learning machine—Based on particle swarm optimization. Advances in Neural Networks—ISNN 2006.

[18] Zong W., Huang G.B., Chen Y. (2013). Weighted extreme learning machine for imbalance learning. Neurocomputing.

[19] Mehrabian A., Lucas C. (2006). A novel numerical optimization algorithm inspired from weed colonization. Ecol. Informatics.

[20] Raslan A.F., Ali A.F., Darwish A. (2020). 1—Swarm intelligence algorithms and their applications in Internet of Things. Swarm Intelligence for Resource Management in Internet of Things.

[21] Dorigo M., Birattari M. (2010). Ant Colony Optimization. Encyclopedia of Machine Learning.

[22] Kennedy J., Eberhart R. Particle swarm optimization. Proceedings of the ICNN’95—International Conference on Neural Networks.

[23] Karaboga D., Basturk B. (2008). On the performance of artificial bee colony (ABC) algorithm. Appl. Soft Comput..

[24] Yang X.S., Watanabe O., Zeugmann T. (2009). Firefly algorithms for multimodal optimization. Stochastic Algorithms: Foundations and Applications.

[25] Gandomi A.H., Yang X.S., Alavi A.H. (2013). Cuckoo search algorithm: A metaheuristic approach to solve structural optimization problems. Eng. Comput..

[26] Yang X., Gandomi A.H. (2012). Bat algorithm: A novel approach for global engineering optimization. Eng. Comput..

[27] Mirjalili S., Lewis A. (2016). The Whale Optimization Algorithm. Adv. Eng. Softw..

[28] Wang G.G., Deb S., Coelho L.d.S. Elephant Herding Optimization. Proceedings of the 2015 3rd International Symposium on Computational and Business Intelligence (ISCBI).

[29] Mucherino A., Seref O. (2007). Monkey search: A novel metaheuristic search for global optimization. AIP Conf. Proc..

[30] Mirjalili S., Mirjalili S.M., Lewis A. (2014). Grey Wolf Optimizer. Adv. Eng. Softw..

[31] Yang X.S. (2012). Flower pollination algorithm for global optimization. Unconventional Computation and Natural Computation.

[32] Feng Y., Deb S., Wang G.G., Alavi A.H. (2021). Monarch butterfly optimization: A comprehensive review. Expert Syst. Appl..

[33] Li S., Chen H., Wang M., Heidari A.A., Mirjalili S. (2020). Slime mould algorithm: A new method for stochastic optimization. Future Gener. Comput. Syst..

[34] Wang G.G. (2018). Moth search algorithm: A bio-inspired metaheuristic algorithm for global optimization problems. Memetic Comput..

[35] Yang Y., Chen H., Heidari A.A., Gandomi A.H. (2021). Hunger games search: Visions, conception, implementation, deep analysis, perspectives, and towards performance shifts. Expert Syst. Appl..

[36] Tu J., Chen H., Wang M., Gandomi A.H. (2021). The Colony Predation Algorithm. J. Bionic Eng..

[37] Bezdan T., Petrovic A., Zivkovic M., Strumberger I., Devi V.K., Bacanin N. (2021). Current Best Opposition-Based Learning Salp Swarm Algorithm for Global Numerical Optimization. Proceedings of the 2021 Zooming Innovation in Consumer Technologies Conference (ZINC).

[38] Bezdan T., Zivkovic M., Tuba E., Strumberger I., Bacanin N., Tuba M. (2020). Multi-objective Task Scheduling in Cloud Computing Environment by Hybridized Bat Algorithm. Proceedings of the International Conference on Intelligent and Fuzzy Systems.

[39] Bacanin N., Bezdan T., Tuba E., Strumberger I., Tuba M., Zivkovic M. (2019). Task scheduling in cloud computing environment by grey wolf optimizer. Proceedings of the 2019 27th Telecommunications Forum (TELFOR).

[40] Bacanin N., Zivkovic M., Bezdan T., Venkatachalam K., Abouhawwash M. (2022). Modified firefly algorithm for workflow scheduling in cloud-edge environment. Neural Comput. Appl..

[41] Bacanin N., Sarac M., Budimirovic N., Zivkovic M., AlZubi A.A., Bashir A.K. (2022). Smart wireless health care system using graph LSTM pollution prediction and dragonfly node localization. Sustain. Comput. Infor. Syst..

[42] Zivkovic M., Bacanin N., Tuba E., Strumberger I., Bezdan T., Tuba M. (2020). Wireless Sensor Networks Life Time Optimization Based on the Improved Firefly Algorithm. Proceedings of the 2020 International Wireless Communications and Mobile Computing (IWCMC).

[43] Bacanin N., Tuba E., Zivkovic M., Strumberger I., Tuba M. (2019). Whale Optimization Algorithm with Exploratory Move for Wireless Sensor Networks Localization. Proceedings of the International Conference on Hybrid Intelligent Systems.

[44] Zivkovic M., Bacanin N., Zivkovic T., Strumberger I., Tuba E., Tuba M. (2020). Enhanced Grey Wolf Algorithm for Energy Efficient Wireless Sensor Networks. Proceedings of the 2020 Zooming Innovation in Consumer Technologies Conference (ZINC).

[45] Bacanin N., Stoean R., Zivkovic M., Petrovic A., Rashid T.A., Bezdan T. (2021). Performance of a Novel Chaotic Firefly Algorithm with Enhanced Exploration for Tackling Global Optimization Problems: Application for Dropout Regularization. Mathematics.

[46] Strumberger I., Tuba E., Bacanin N., Zivkovic M., Beko M., Tuba M. (2019). Designing convolutional neural network architecture by the firefly algorithm. Proceedings of the 2019 International Young Engineers Forum (YEF-ECE).

[47] Milosevic S., Bezdan T., Zivkovic M., Bacanin N., Strumberger I., Tuba M. (2021). Feed-Forward Neural Network Training by Hybrid Bat Algorithm. Modelling and Development of Intelligent Systems, Proceedings of the 7th International Conference, MDIS 2020, Sibiu, Romania, 22–24 October 2020.

[48] Bezdan T., Stoean C., Naamany A.A., Bacanin N., Rashid T.A., Zivkovic M., Venkatachalam K. (2021). Hybrid Fruit-Fly Optimization Algorithm with K-Means for Text Document Clustering. Mathematics.

[49] Cuk A., Bezdan T., Bacanin N., Zivkovic M., Venkatachalam K., Rashid T.A., Devi V.K. (2021). Feedforward multi-layer perceptron training by hybridized method between genetic algorithm and artificial bee colony. Data Science and Data Analytics: Opportunities and Challenges.

[50] Stoean R. (2020). Analysis on the potential of an EA–surrogate modelling tandem for deep learning parametrization: An example for cancer classification from medical images. Neural Comput. Appl..

[51] Bacanin N., Bezdan T., Zivkovic M., Chhabra A. (2022). Weight optimization in artificial neural network training by improved monarch butterfly algorithm. Mobile Computing and Sustainable Informatics.

[52] Gajic L., Cvetnic D., Zivkovic M., Bezdan T., Bacanin N., Milosevic S. (2021). Multi-layer perceptron training using hybridized bat algorithm. Computational Vision and Bio-Inspired Computing.

[53] Bacanin N., Alhazmi K., Zivkovic M., Venkatachalam K., Bezdan T., Nebhen J. (2022). Training Multi-Layer Perceptron with Enhanced Brain Storm Optimization Metaheuristics. Comput. Mater. Contin..

[54] Jnr E.O.N., Ziggah Y.Y., Relvas S. (2021). Hybrid ensemble intelligent model based on wavelet transform, swarm intelligence and artificial neural network for electricity demand forecasting. Sustain. Cities Soc..

[55] Bacanin N., Bezdan T., Venkatachalam K., Zivkovic M., Strumberger I., Abouhawwash M., Ahmed A. (2021). Artificial Neural Networks Hidden Unit and Weight Connection Optimization by Quasi-Refection-Based Learning Artificial Bee Colony Algorithm. IEEE Access.

[56] Bacanin N., Zivkovic M., Bezdan T., Cvetnic D., Gajic L. (2022). Dimensionality Reduction Using Hybrid Brainstorm Optimization Algorithm. Proceedings of the International Conference on Data Science and Applications.

[57] Latha R.S., Saravana Balaji B., Bacanin N., Strumberger I., Zivkovic M., Kabiljo M. (2022). Feature Selection Using Grey Wolf Optimization with Random Differential Grouping. Comput. Syst. Sci. Eng..

[58] Zivkovic M., Stoean C., Chhabra A., Budimirovic N., Petrovic A., Bacanin N. (2022). Novel Improved Salp Swarm Algorithm: An Application for Feature Selection. Sensors.

[59] Bacanin N., Petrovic A., Zivkovic M., Bezdan T., Antonijevic M. (2021). Feature Selection in Machine Learning by Hybrid Sine Cosine Metaheuristics. Proceedings of the International Conference on Advances in Computing and Data Sciences.

[60] Salb M., Zivkovic M., Bacanin N., Chhabra A., Suresh M. (2022). Support vector machine performance improvements for cryptocurrency value forecasting by enhanced sine cosine algorithm. Computer Vision and Robotics.

[61] Bezdan T., Zivkovic M., Tuba E., Strumberger I., Bacanin N., Tuba M. (2020). Glioma Brain Tumor Grade Classification from MRI Using Convolutional Neural Networks Designed by Modified FA. Proceedings of the International Conference on Intelligent and Fuzzy Systems.

[62] Bezdan T., Milosevic S., Venkatachalam K., Zivkovic M., Bacanin N., Strumberger I. (2021). Optimizing Convolutional Neural Network by Hybridized Elephant Herding Optimization Algorithm for Magnetic Resonance Image Classification of Glioma Brain Tumor Grade. Proceedings of the 2021 Zooming Innovation in Consumer Technologies Conference (ZINC).

[63] Basha J., Bacanin N., Vukobrat N., Zivkovic M., Venkatachalam K., Hubálovskỳ S., Trojovskỳ P. (2021). Chaotic Harris Hawks Optimization with Quasi-Reflection-Based Learning: An Application to Enhance CNN Design. Sensors.

[64] Tair M., Bacanin N., Zivkovic M., Venkatachalam K. (2022). A Chaotic Oppositional Whale Optimisation Algorithm with Firefly Search for Medical Diagnostics. Comput. Mater. Contin..

[65] Zivkovic M., Bacanin N., Venkatachalam K., Nayyar A., Djordjevic A., Strumberger I., Al-Turjman F. (2021). COVID-19 cases prediction by using hybrid machine learning and beetle antennae search approach. Sustain. Cities Soc..

[66] Zivkovic M., Venkatachalam K., Bacanin N., Djordjevic A., Antonijevic M., Strumberger I., Rashid T.A. (2021). Hybrid Genetic Algorithm and Machine Learning Method for COVID-19 Cases Prediction. Proceedings of the International Conference on Sustainable Expert Systems: ICSES 2020.

[67] Zivkovic M., Jovanovic L., Ivanovic M., Krdzic A., Bacanin N., Strumberger I. (2022). Feature selection using modified sine cosine algorithm with COVID-19 dataset. Evolutionary Computing and Mobile Sustainable Networks.

[68] Bui D.T., Ngo P.T.T., Pham T.D., Jaafari A., Minh N.Q., Hoa P.V., Samui P. (2019). A novel hybrid approach based on a swarm intelligence optimized extreme learning machine for flash flood susceptibility mapping. Catena.

[69] Feng Z.k., Niu W.j., Zhang R., Wang S., Cheng C.T. (2019). Operation rule derivation of hydropower reservoir by k-means clustering method and extreme learning machine based on particle swarm optimization. J. Hydrol..

[70] Faris H., Mirjalili S., Aljarah I., Mafarja M., Heidari A.A. (2020). Salp swarm algorithm: Theory, literature review, and application in extreme learning machines. Nature-Inspired Optimizers.

[71] Chen H., Zhang Q., Luo J., Xu Y., Zhang X. (2020). An enhanced bacterial foraging optimization and its application for training kernel extreme learning machine. Appl. Soft Comput..

[72] Karaboga D. (2005). An Idea Based on Honey Bee Swarm for Numerical Optimization.

[73] Tuba M., Bacanin N. (2014). Artificial Bee Colony Algorithm Hybridized with Firefly Algorithm for Cardinality Constrained Mean-Variance Portfolio Selection Problem. Appl. Math. Inf. Sci..

[74] Mirjalili S. (2016). SCA: A Sine Cosine Algorithm for solving optimization problems. Knowl. Based Syst..

[75] Bačanin Dzakula N. (2015). Unapređenje Hibridizacijom Metaheuristika Inteligencije Rojeva za Resavanje Problema Globalne Optimizacije. Ph.D. Thesis.

[76] Talbi E.G. (2016). Combining metaheuristics with mathematical programming, constraint programming and machine learning. Ann. Oper. Res..

[77] Bacanin N., Tuba M., Strumberger I. RFID Network Planning by ABC Algorithm Hybridized with Heuristic for Initial Number and Locations of Readers. Proceedings of the 2015 17th UKSim-AMSS International Conference on Modelling and Simulation (UKSim).

[78] Attiya I., Abd Elaziz M., Abualigah L., Nguyen T.N., Abd El-Latif A.A. (2022). An Improved Hybrid Swarm Intelligence for Scheduling IoT Application Tasks in the Cloud. IEEE Trans. Ind. Infor..

[79] Wu X., Li R., Chu C.H., Amoasi R., Liu S. (2022). Managing pharmaceuticals delivery service using a hybrid particle swarm intelligence approach. Ann. Oper. Res..

[80] Bezdan T., Cvetnic D., Gajic L., Zivkovic M., Strumberger I., Bacanin N. Feature Selection by Firefly Algorithm with Improved Initialization Strategy. Proceedings of the 7th Conference on the Engineering of Computer Based Systems.

[81] Caponetto R., Fortuna L., Fazzino S., Xibilia M.G. (2003). Chaotic sequences to improve the performance of evolutionary algorithms. IEEE Trans. Evol. Comput..

[82] Wang M., Chen H. (2020). Chaotic multi-swarm whale optimizer boosted support vector machine for medical diagnosis. Appl. Soft Comput..

[83] Kose U. (2018). An ant-lion optimizer-trained artificial neural network system for chaotic electroencephalogram (EEG) prediction. Appl. Sci..

[84] Yu H., Zhao N., Wang P., Chen H., Li C. (2020). Chaos-enhanced synchronized bat optimizer. Appl. Math. Model..

[85] Rahnamayan S., Tizhoosh H.R., Salama M.M.A. Quasi-oppositional Differential Evolution. Proceedings of the 2007 IEEE Congress on Evolutionary Computation.

[86] Yang X.S., He X. (2013). Firefly algorithm: Recent advances and applications. Int. J. Swarm Intell..

[87] Yang X.S. (2011). Bat algorithm for multi-objective optimisation. Int. J. Bio-Inspired Comput..

[88] Heidari A.A., Mirjalili S., Faris H., Aljarah I., Mafarja M., Chen H. (2019). Harris hawks optimization: Algorithm and applications. Future Gener. Comput. Syst..

[89] Friedman M. (1937). The use of ranks to avoid the assumption of normality implicit in the analysis of variance. J. Am. Stat. Assoc..

[90] Friedman M. (1940). A comparison of alternative tests of significance for the problem of m rankings. Ann. Math. Stat..

[91] Tavallaee M., Bagheri E., Lu W., Ghorbani A.A. (2009). A detailed analysis of the KDD CUP 99 data set. Proceedings of the 2009 IEEE Symposium on Computational Intelligence for Security and Defense Applications.

[92] Dhanabal L., Shantharajah S. (2015). A study on NSL-KDD dataset for intrusion detection system based on classification algorithms. Int. J. Adv. Res. Comput. Commun. Eng..

[93] Protić D.D. (2018). Review of KDD Cup’99, NSL-KDD and Kyoto 2006+ datasets. Vojnoteh. Glas..

